# Supplementary material for: Asymmetric cortical projections to striatal direct and indirect pathways distinctly control actions
Source: eLife. 2025 Oct 21;12:RP92992. doi: 10.7554/eLife.92992 (PMC12539805; doi:10.7554/eLife.92992)
Supplement: Figure 1—source data 1. [file elife-92992-fig1-data1.pptx]

## Slide 1
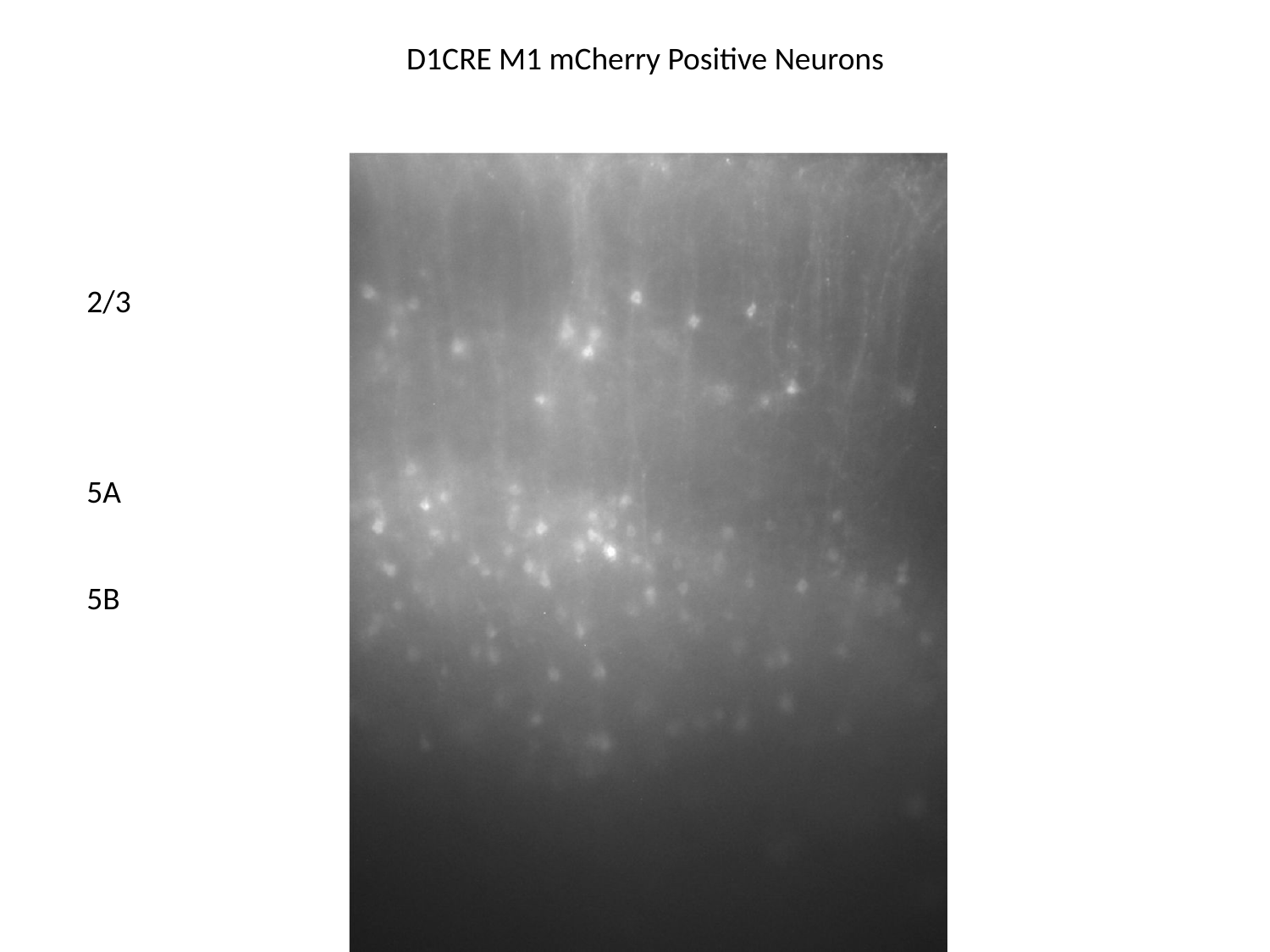

D1CRE M1 mCherry Positive Neurons
2/3
5A
5B

## Slide 2
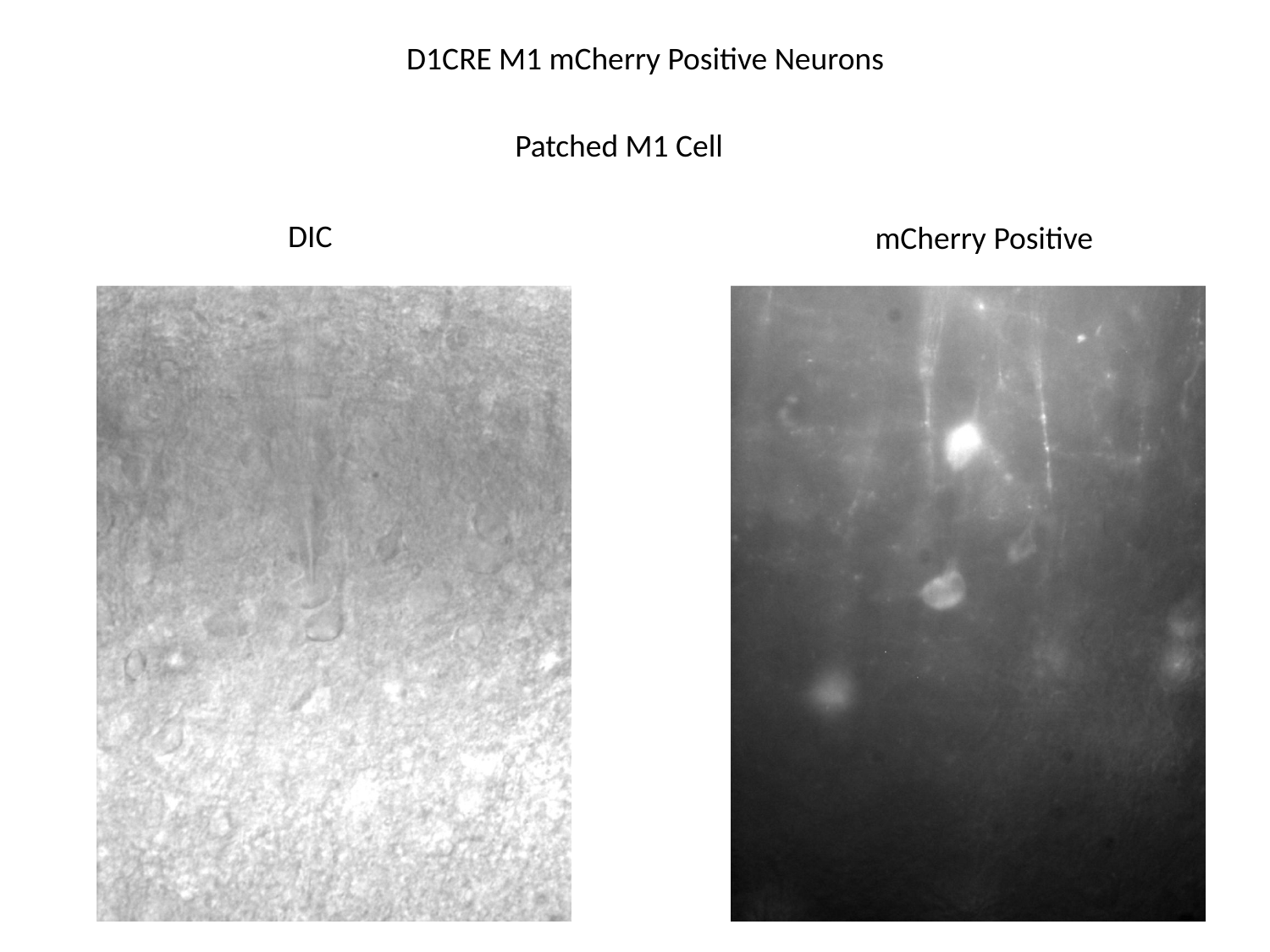

D1CRE M1 mCherry Positive Neurons
Patched M1 Cell
DIC
mCherry Positive

## Slide 3
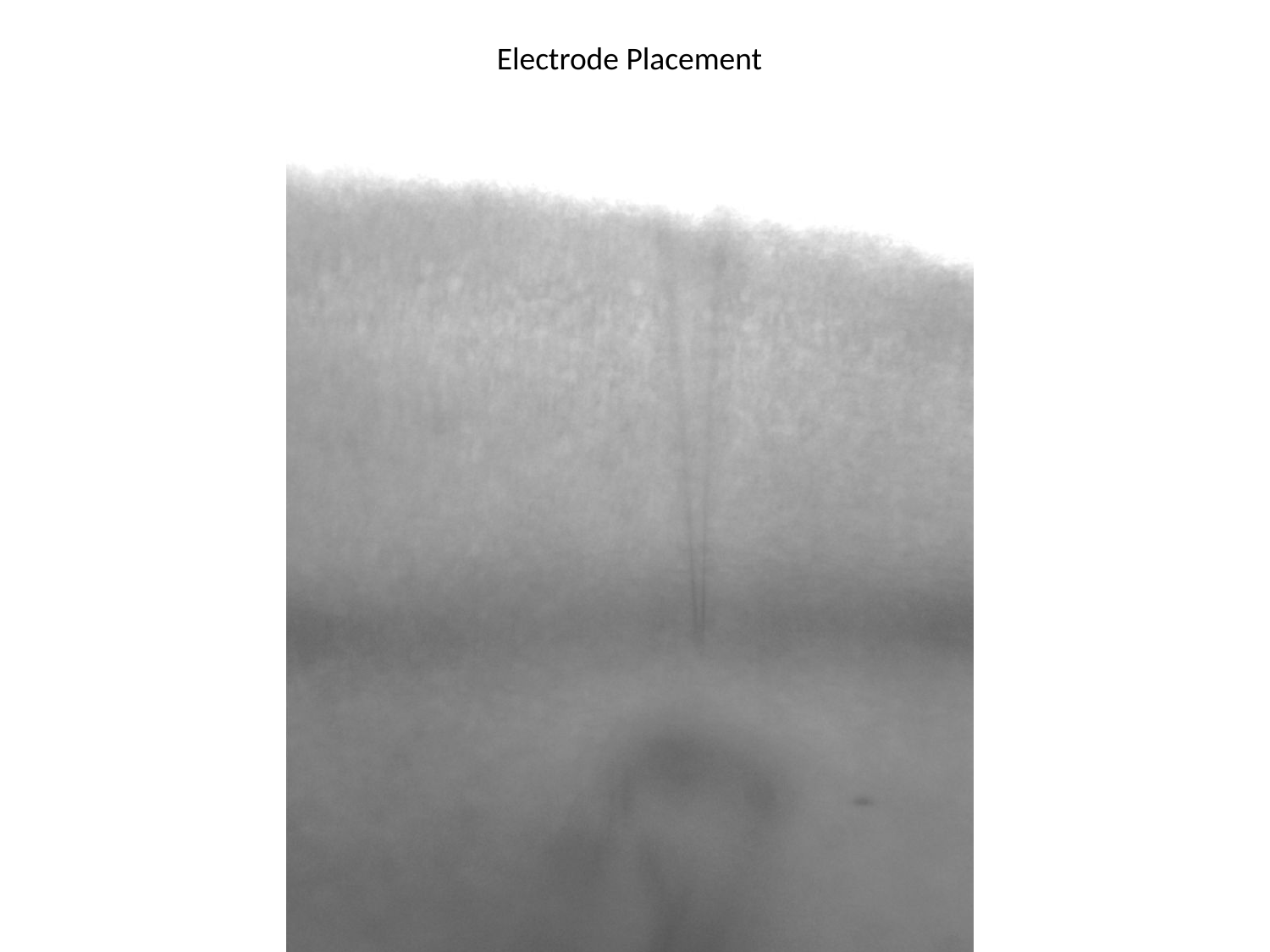

Electrode Placement

## Slide 4
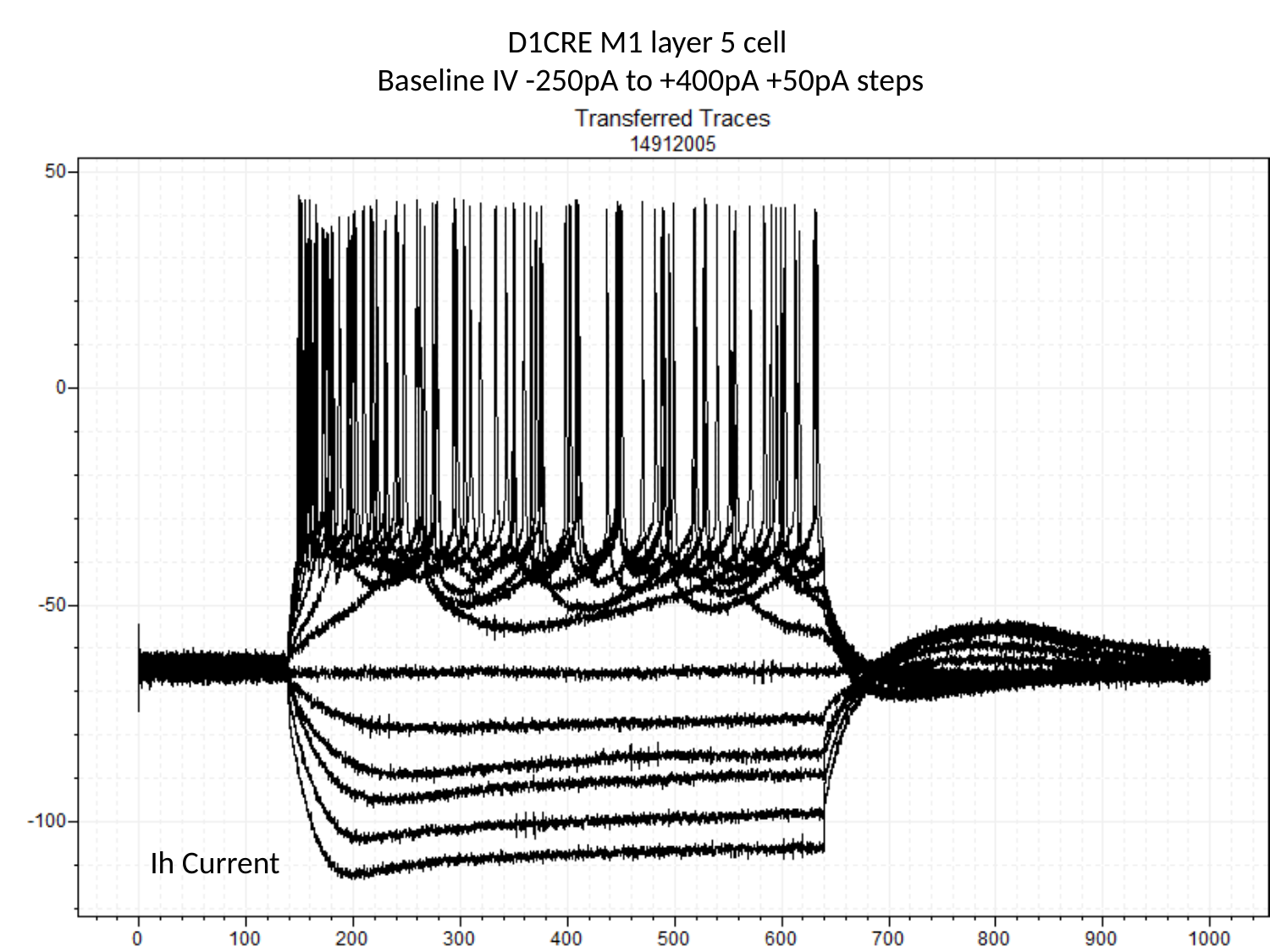

D1CRE M1 layer 5 cell
Baseline IV -250pA to +400pA +50pA steps
Ih Current

## Slide 5
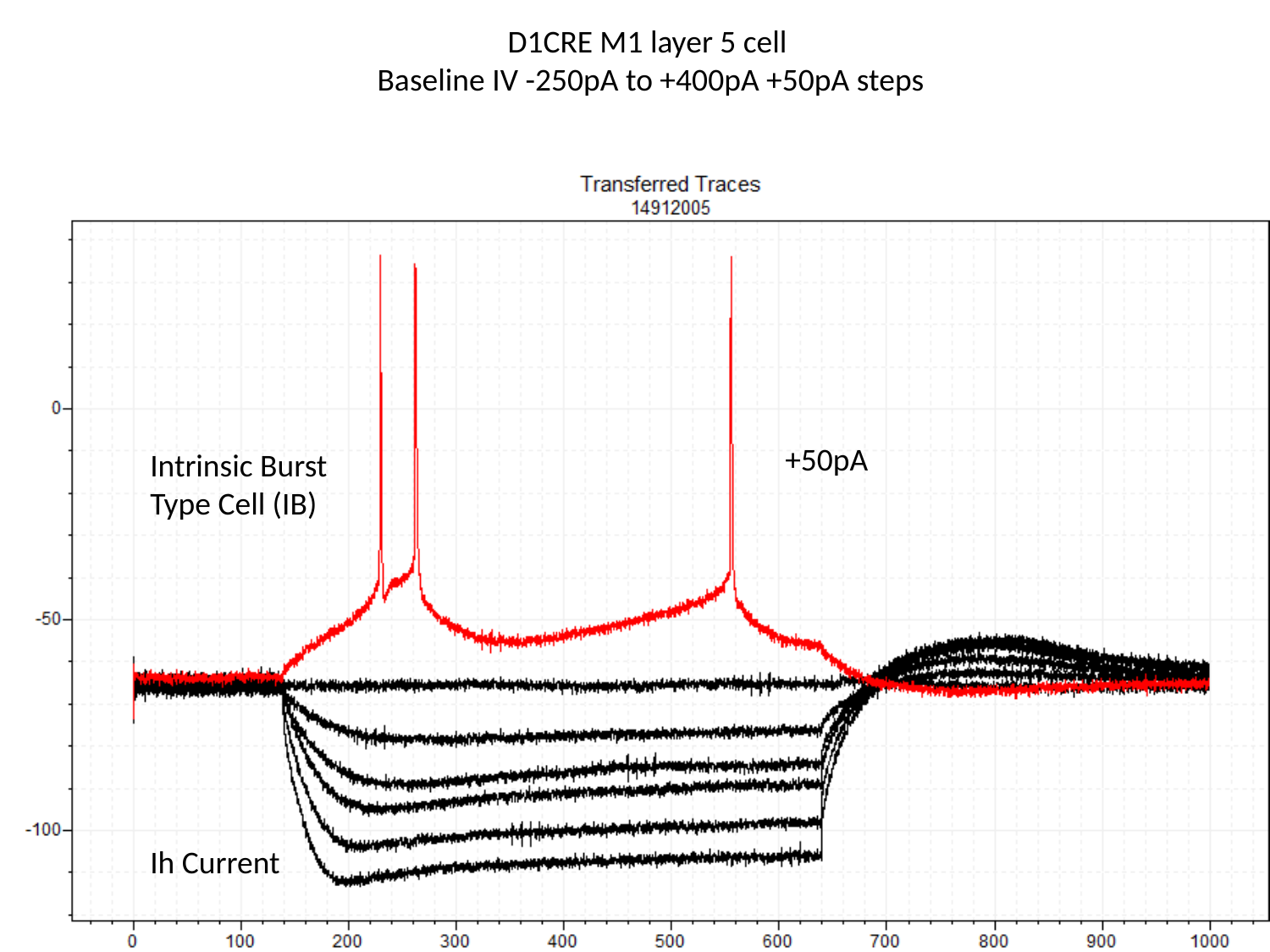

D1CRE M1 layer 5 cell
Baseline IV -250pA to +400pA +50pA steps
+50pA
Intrinsic Burst Type Cell (IB)
Ih Current

## Slide 6
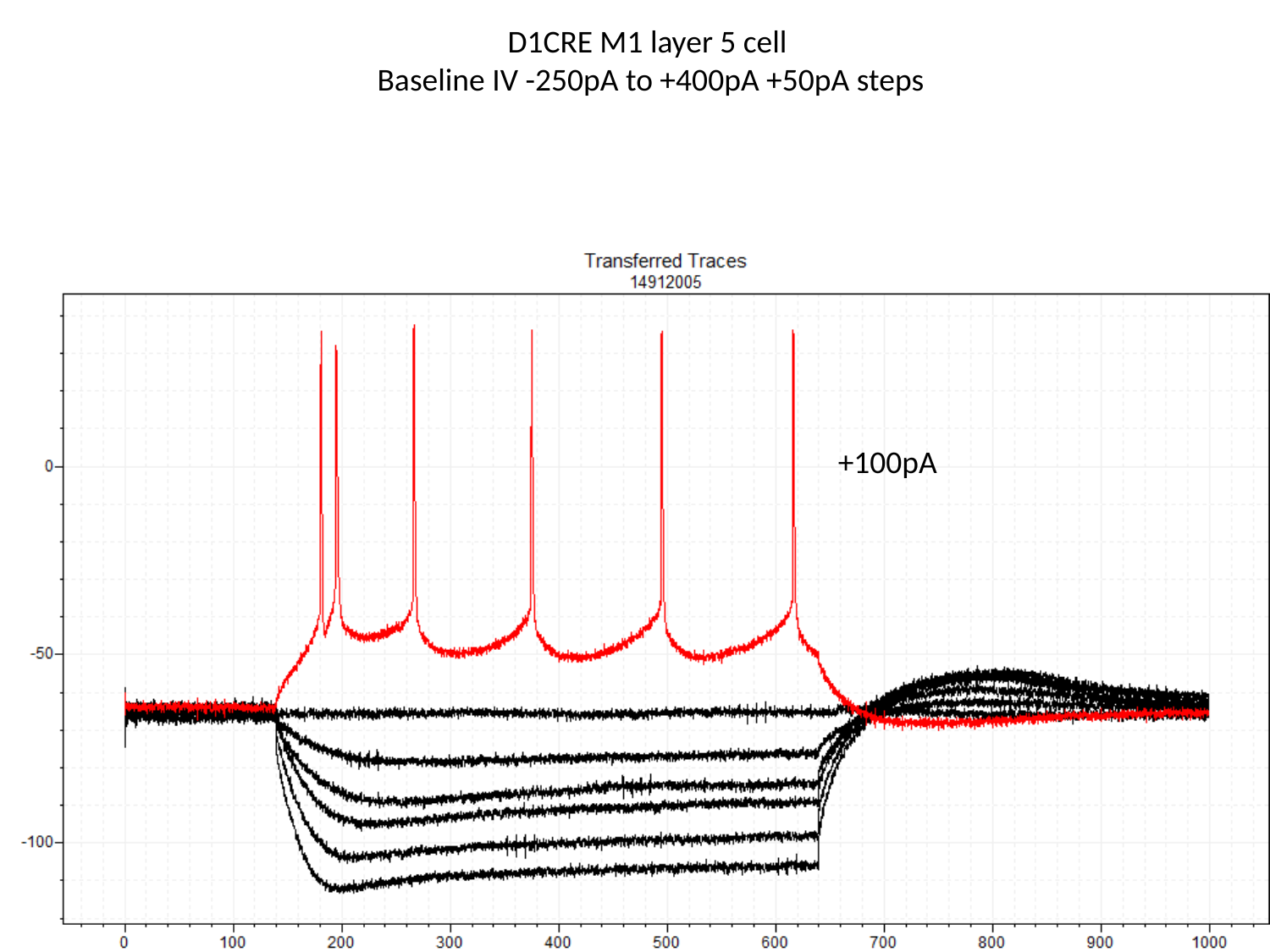

D1CRE M1 layer 5 cell
Baseline IV -250pA to +400pA +50pA steps
+100pA

## Slide 7
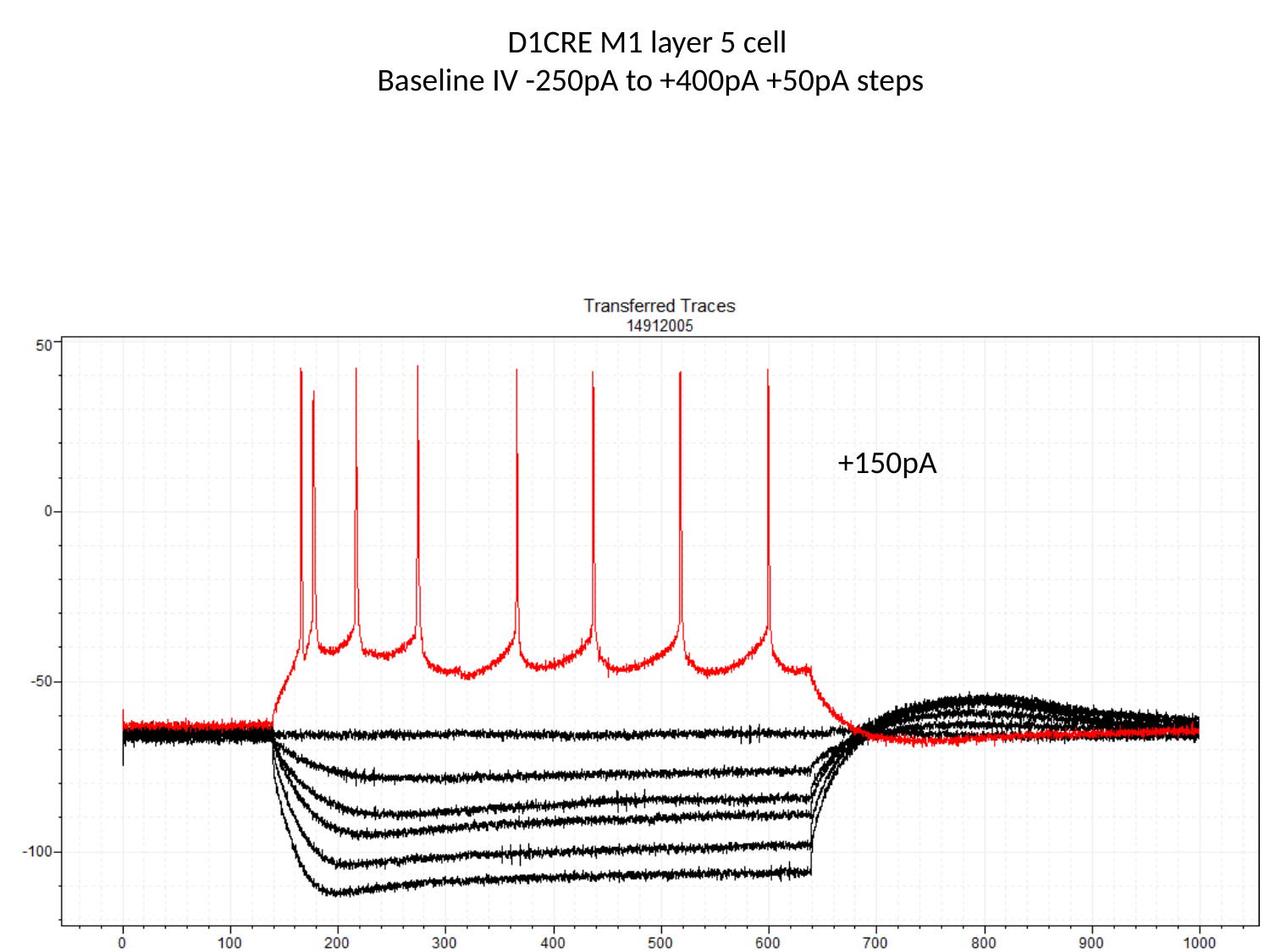

D1CRE M1 layer 5 cell
Baseline IV -250pA to +400pA +50pA steps
+150pA

## Slide 8
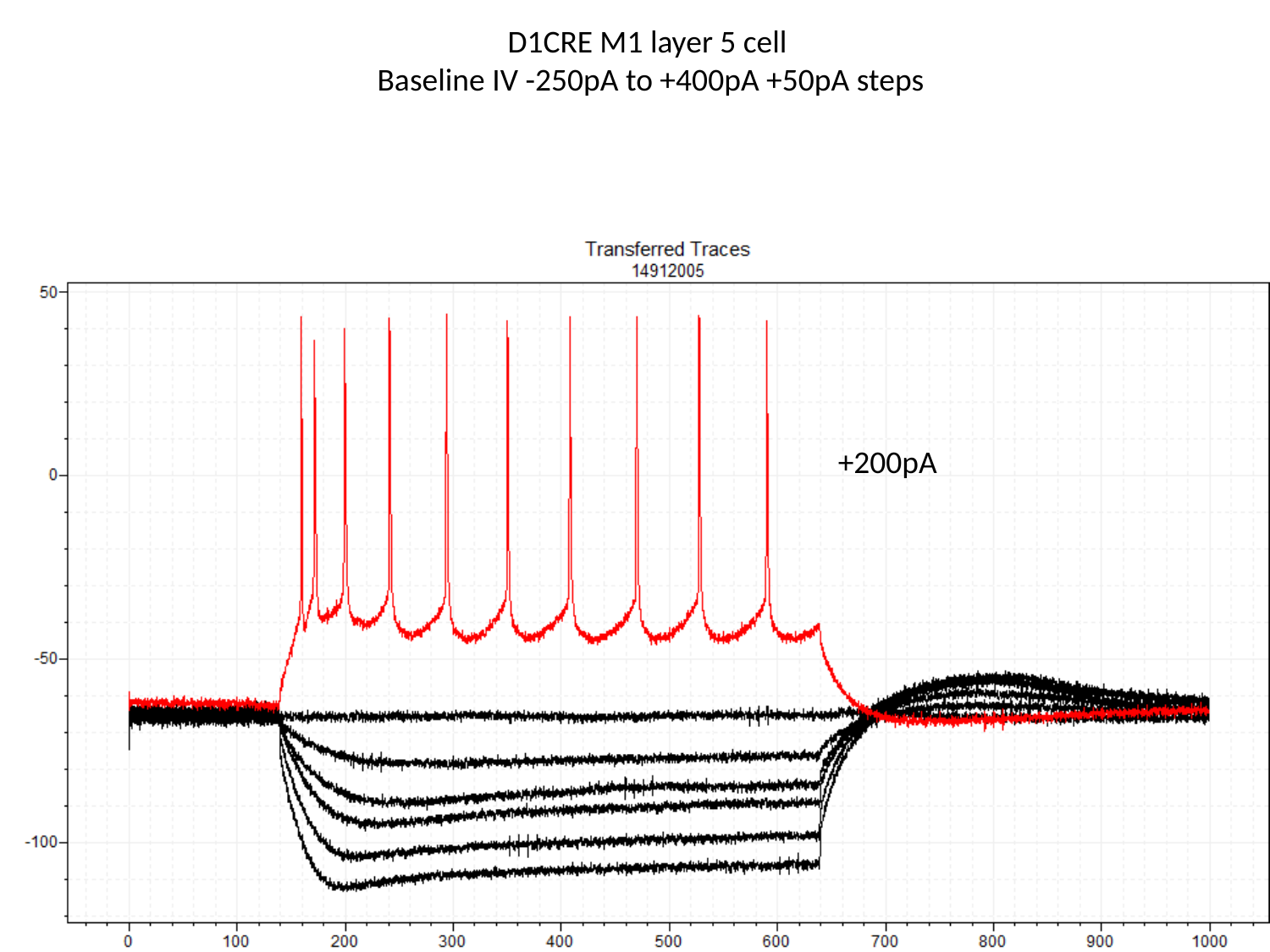

D1CRE M1 layer 5 cell
Baseline IV -250pA to +400pA +50pA steps
+200pA

## Slide 9
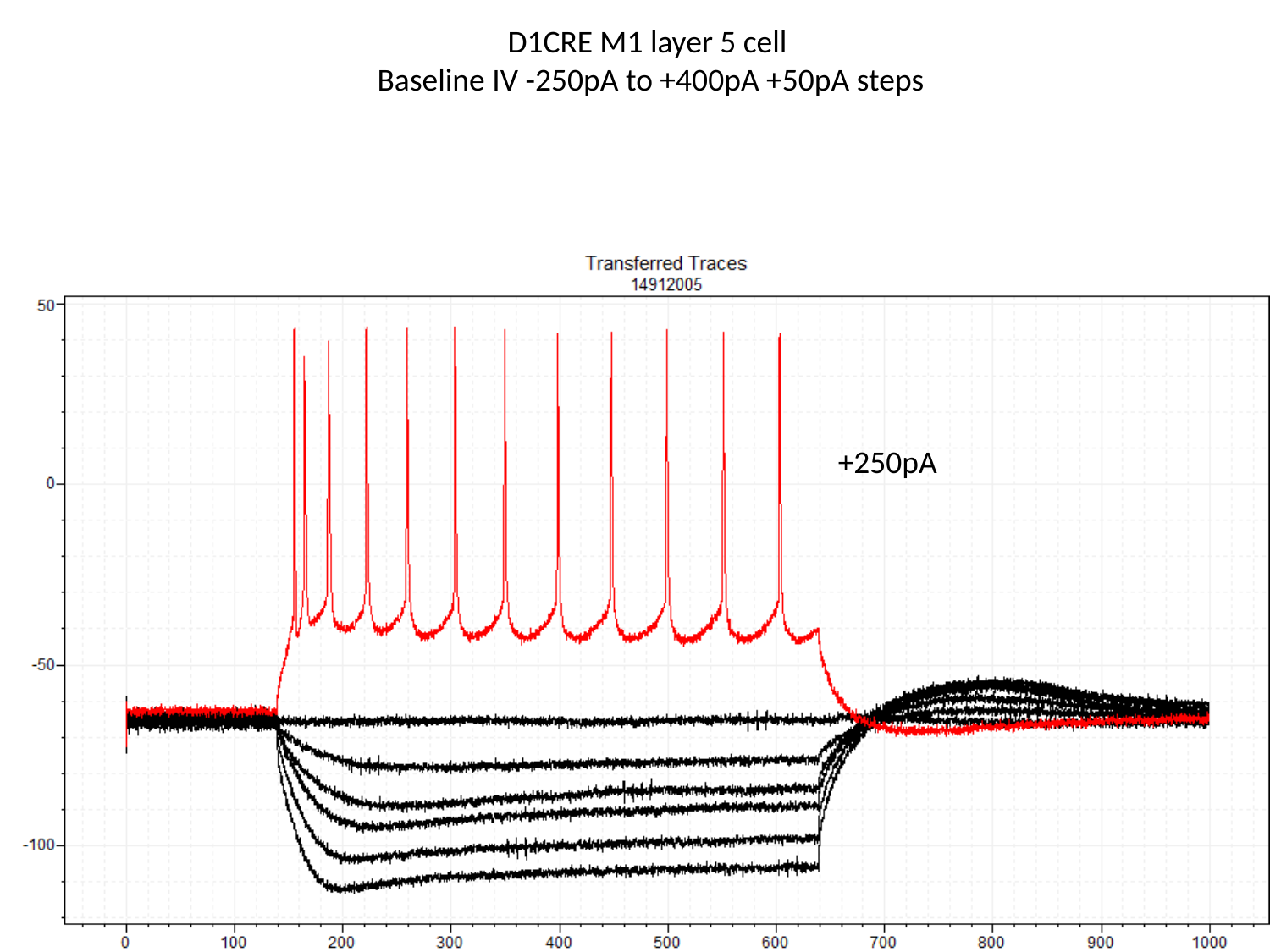

D1CRE M1 layer 5 cell
Baseline IV -250pA to +400pA +50pA steps
+250pA

## Slide 10
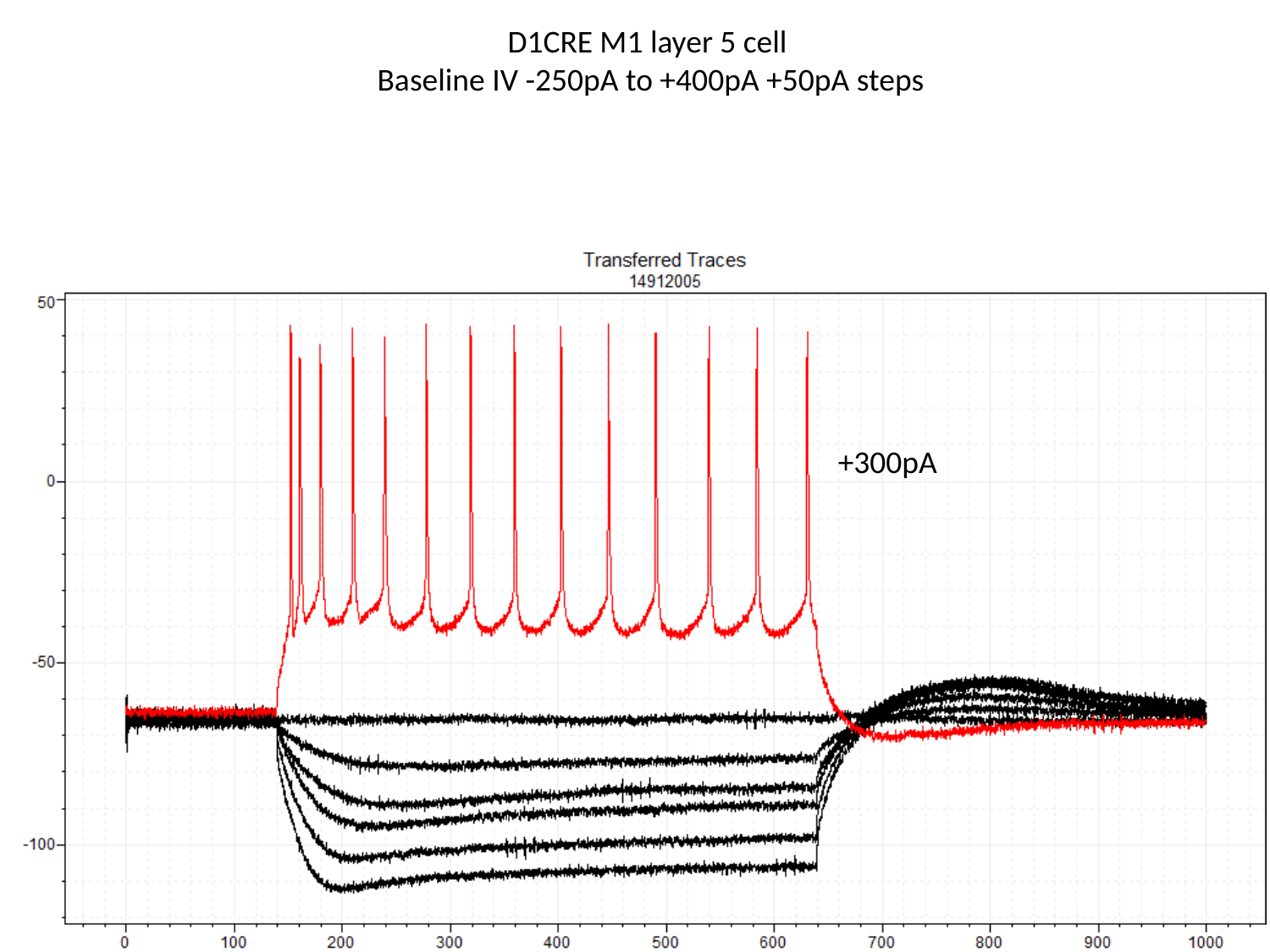

D1CRE M1 layer 5 cell
Baseline IV -250pA to +400pA +50pA steps
+300pA

## Slide 11
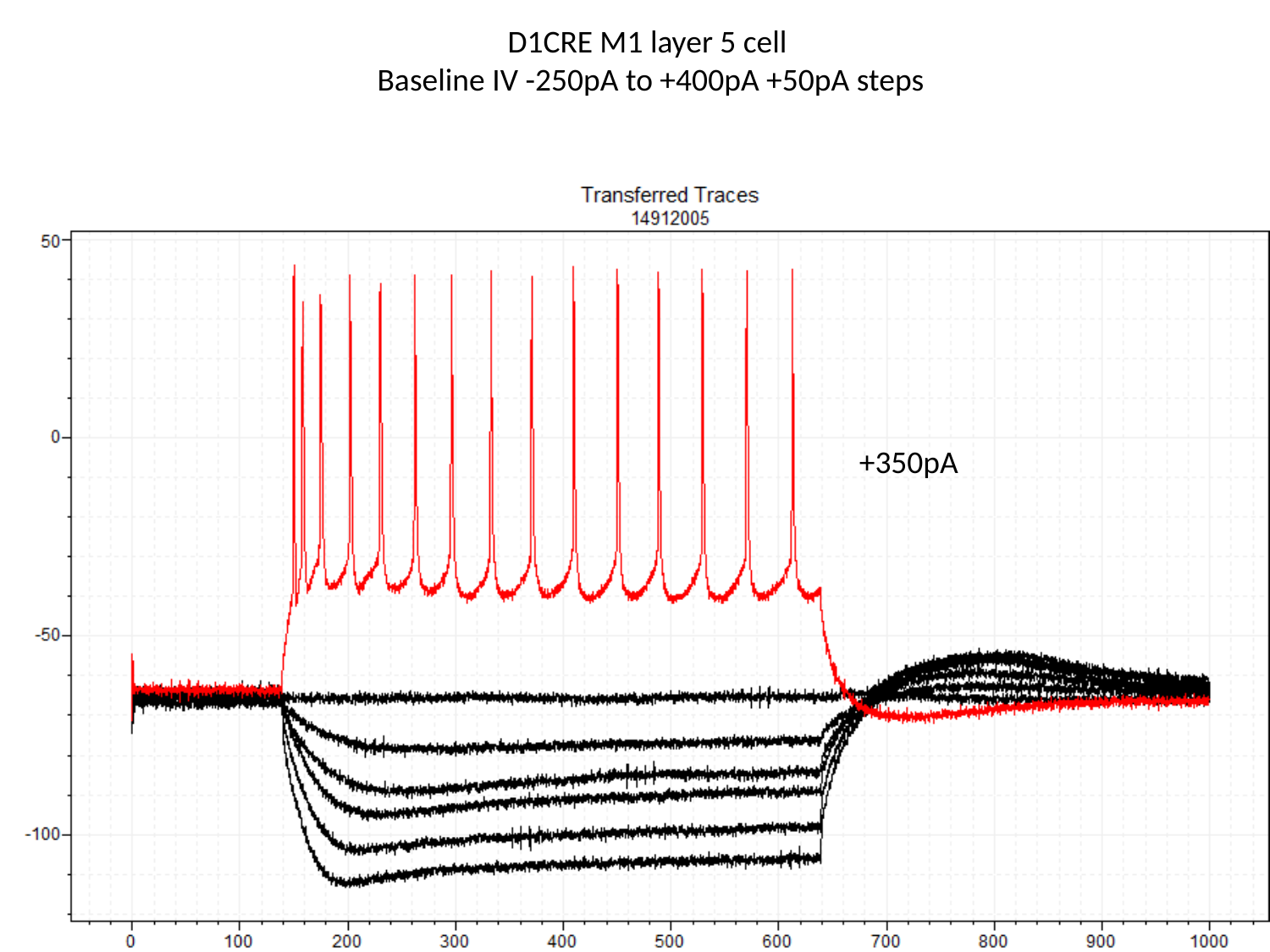

D1CRE M1 layer 5 cell
Baseline IV -250pA to +400pA +50pA steps
+350pA

## Slide 12
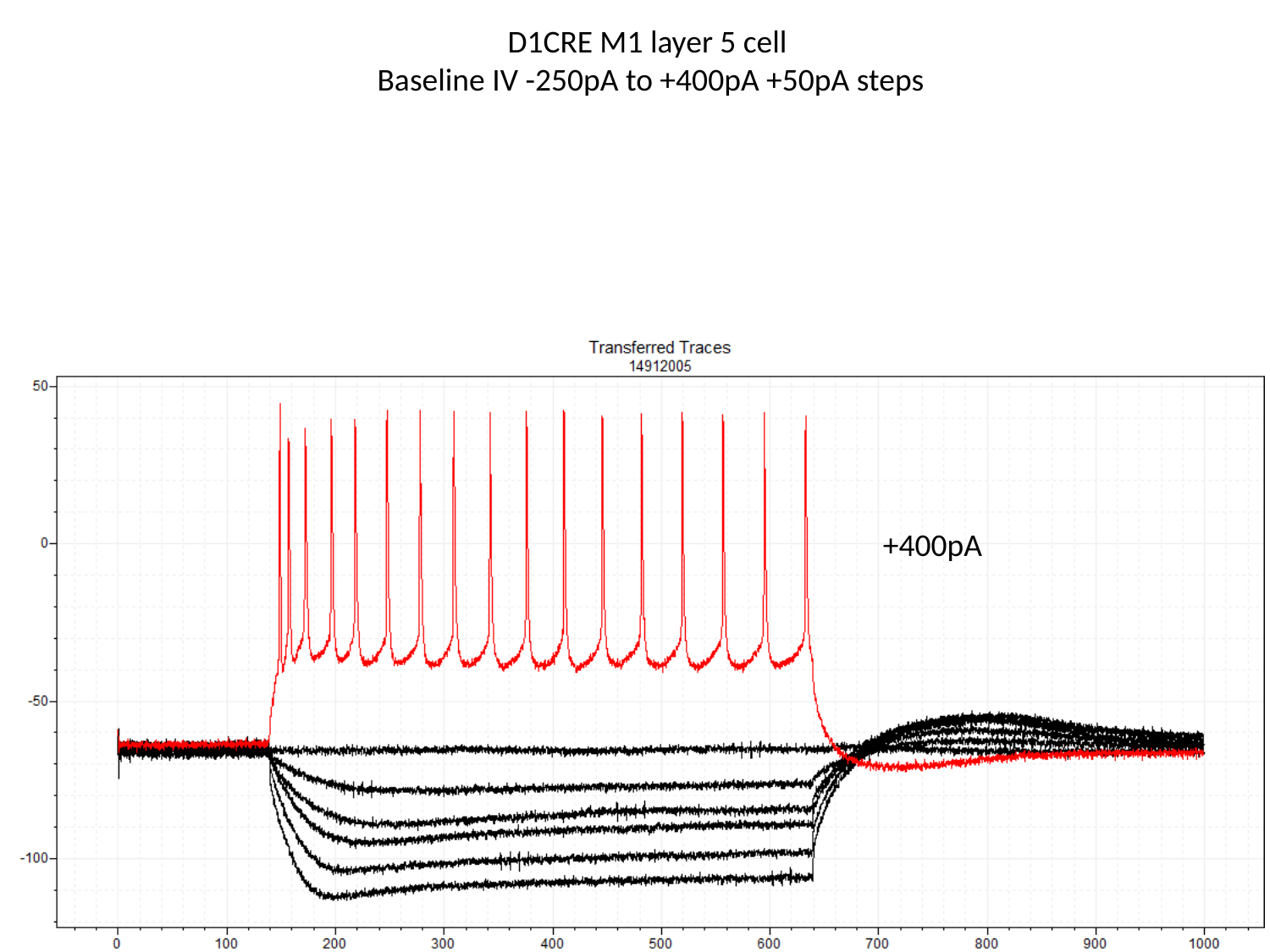

D1CRE M1 layer 5 cell
Baseline IV -250pA to +400pA +50pA steps
+400pA

## Slide 13
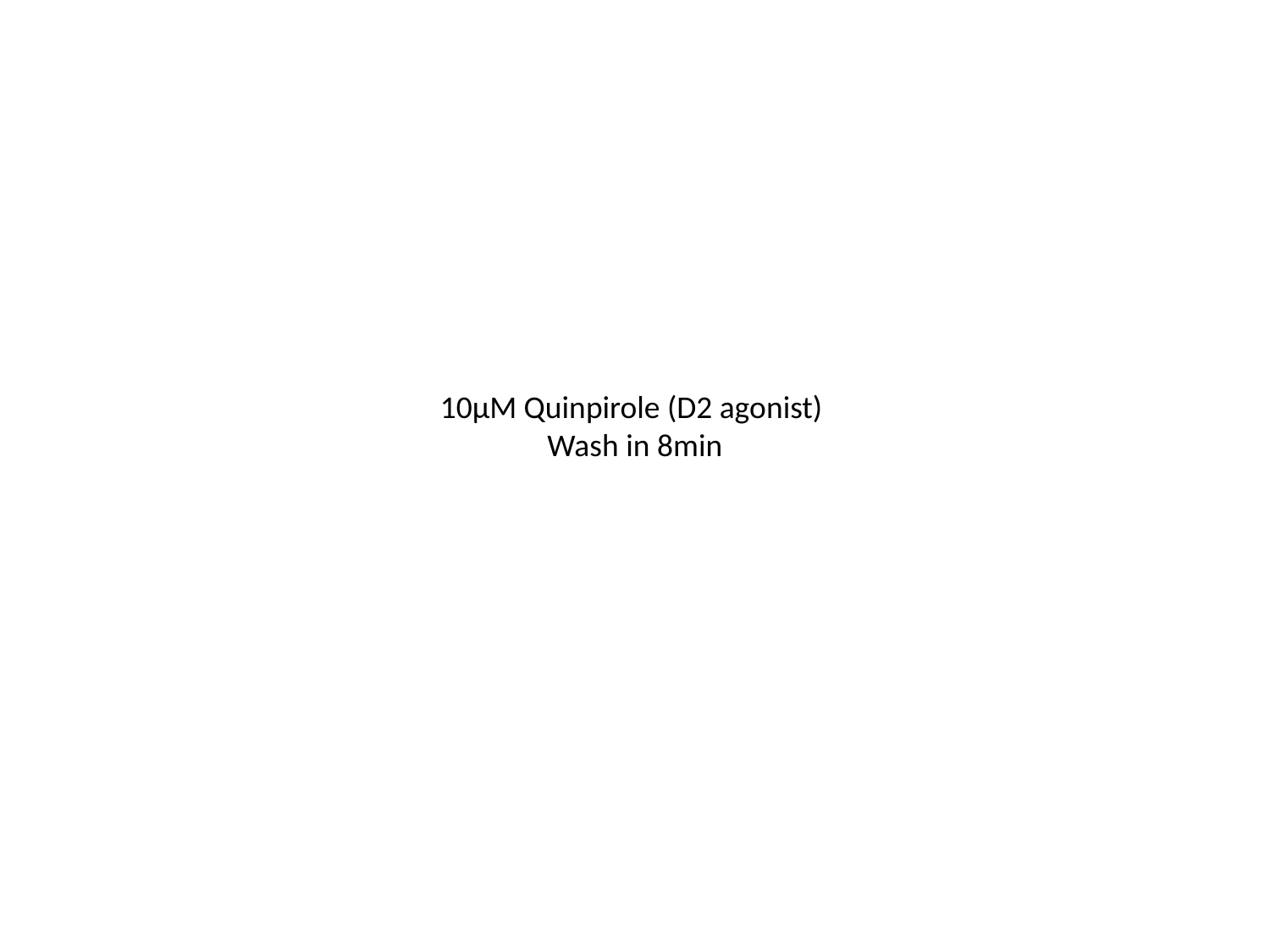

10µM Quinpirole (D2 agonist)
Wash in 8min

## Slide 14
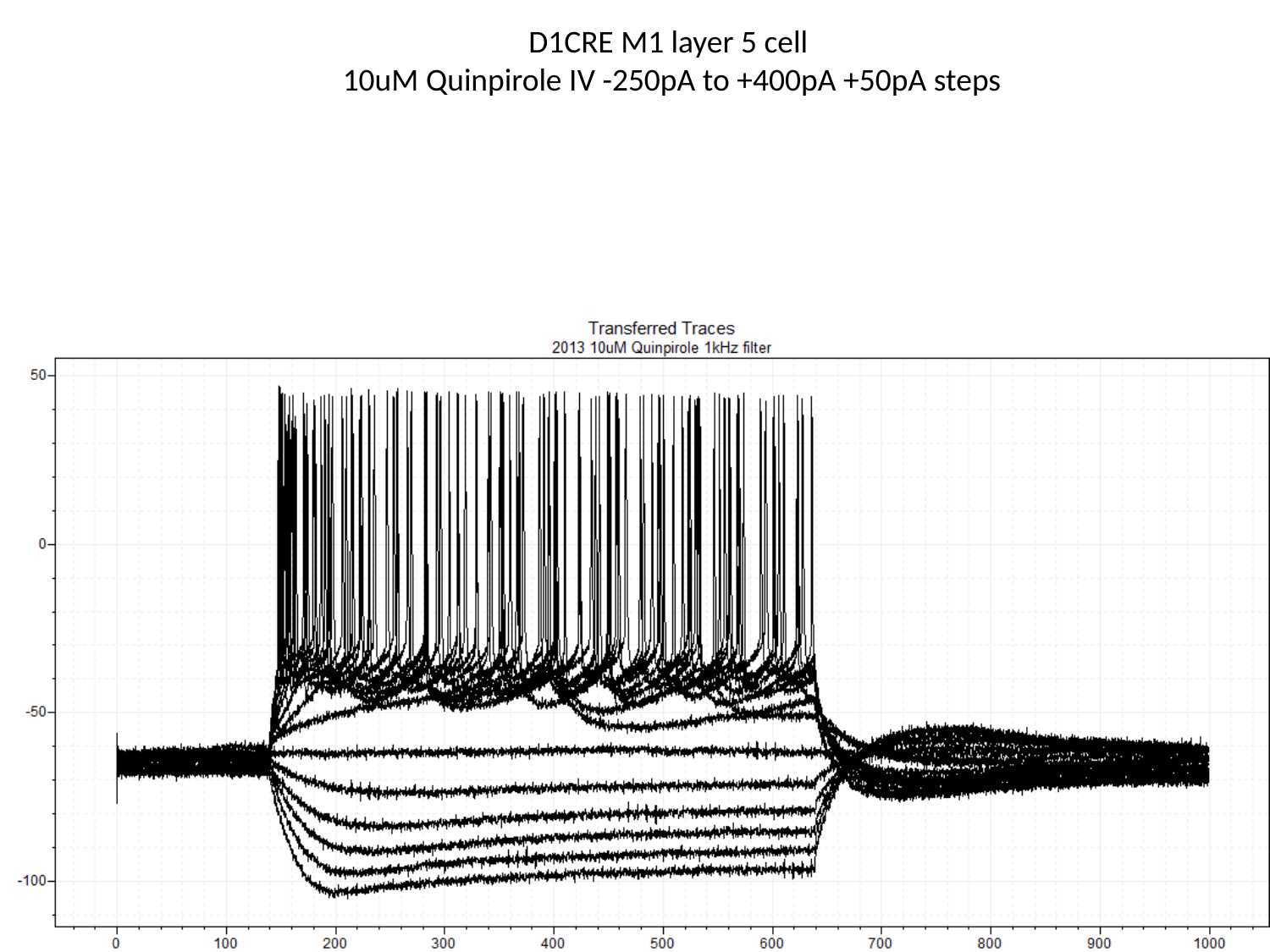

D1CRE M1 layer 5 cell
10uM Quinpirole IV -250pA to +400pA +50pA steps

## Slide 15
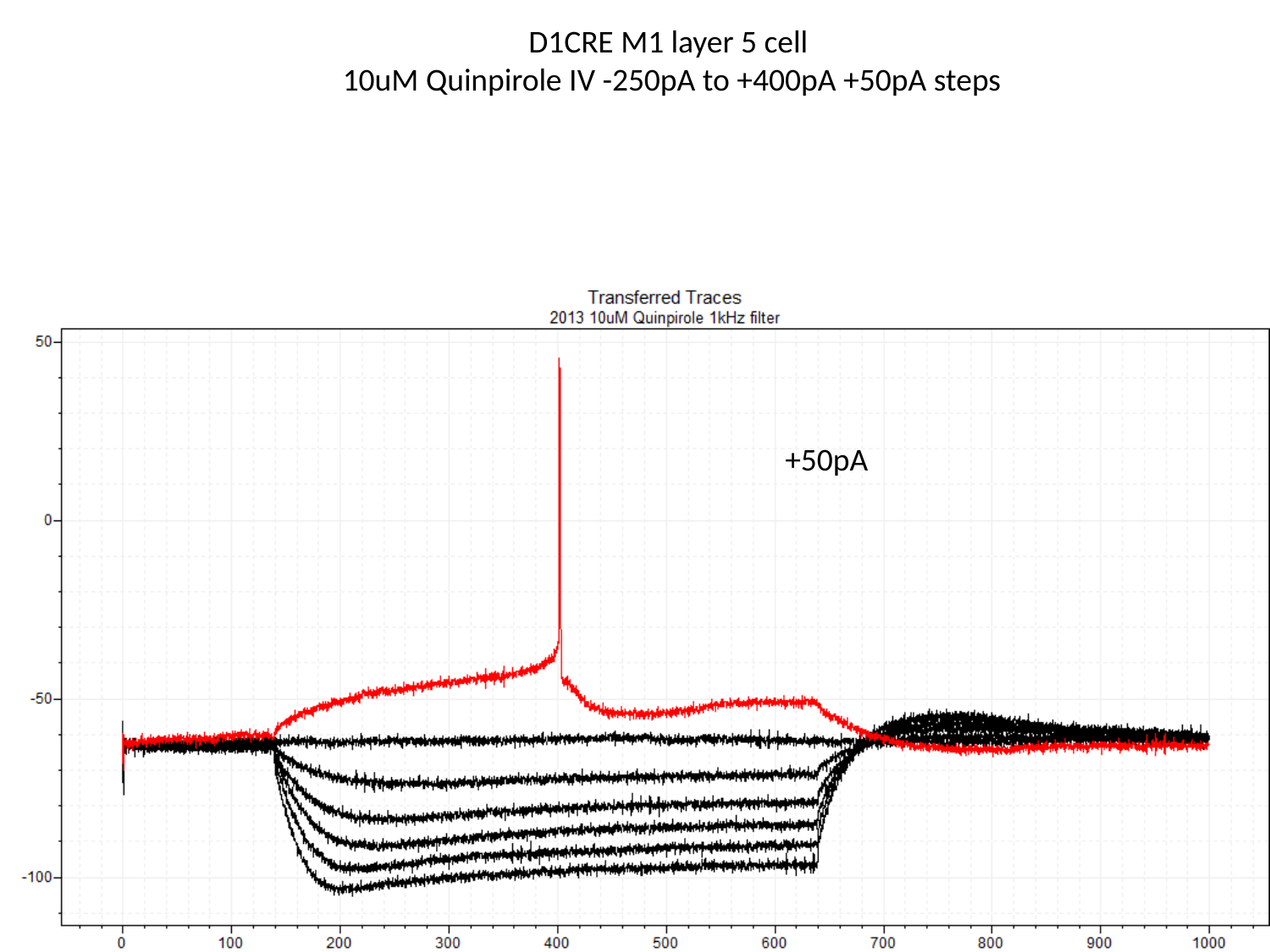

D1CRE M1 layer 5 cell
10uM Quinpirole IV -250pA to +400pA +50pA steps
+50pA

## Slide 16
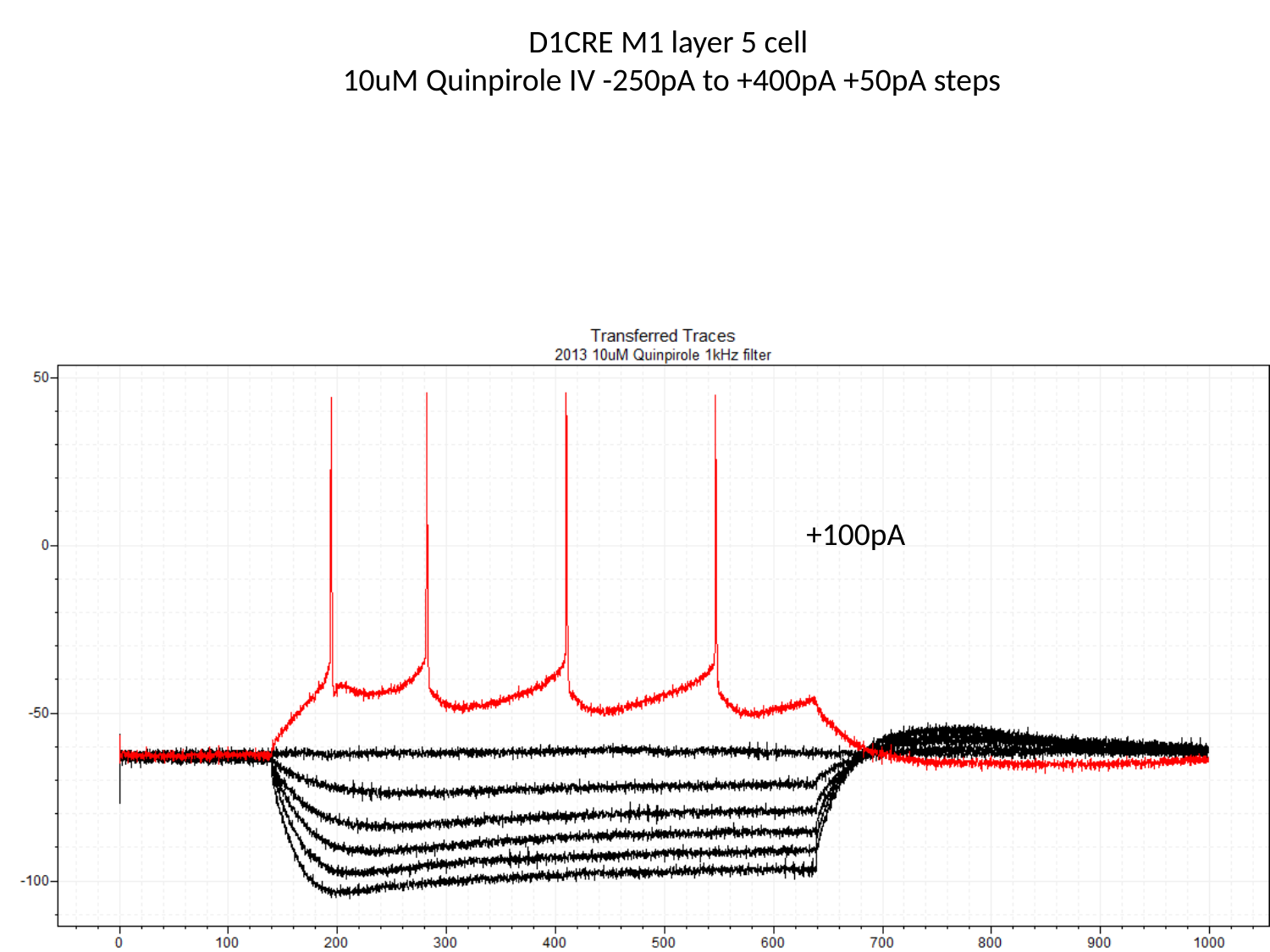

D1CRE M1 layer 5 cell
10uM Quinpirole IV -250pA to +400pA +50pA steps
+100pA

## Slide 17
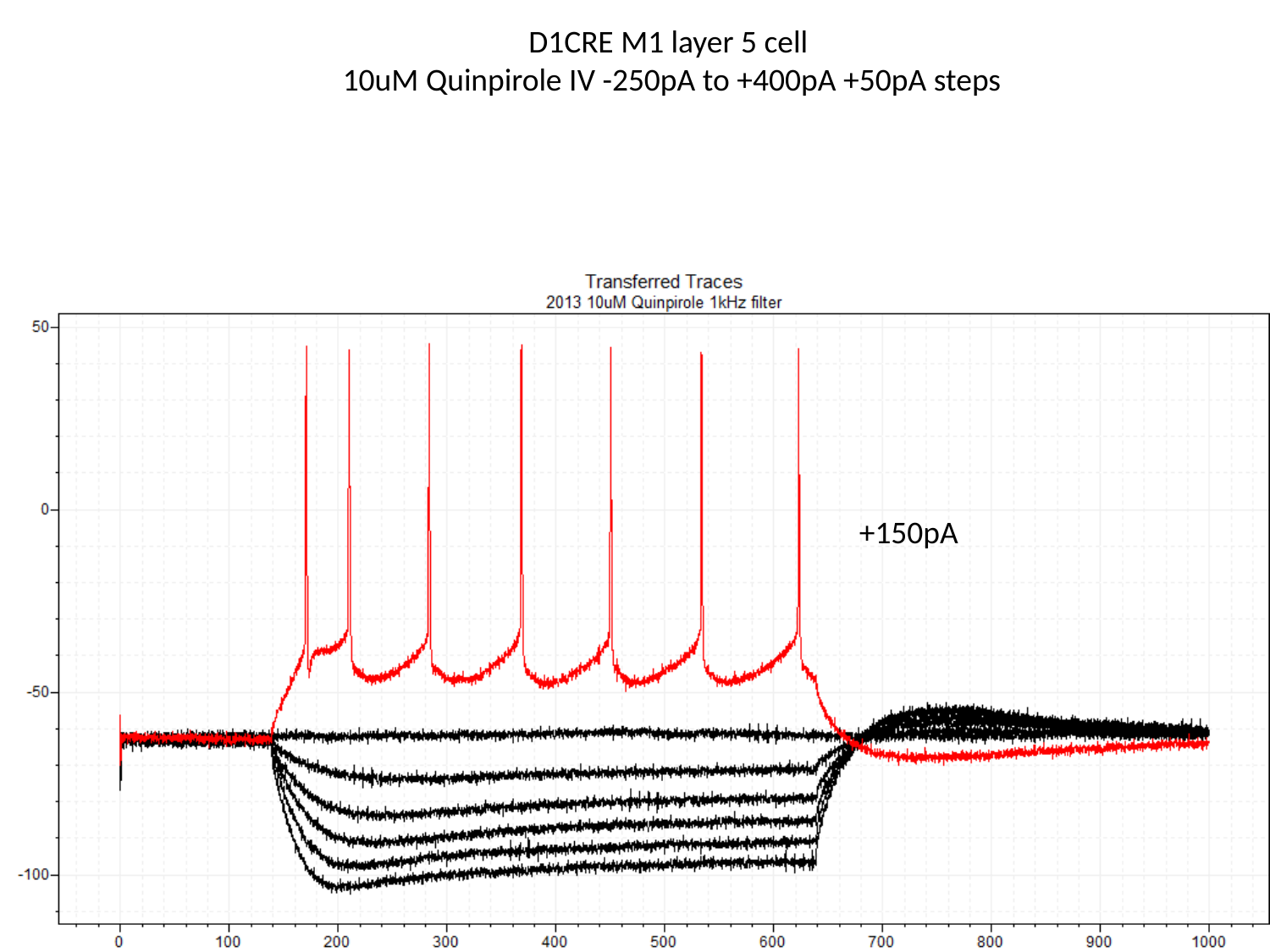

D1CRE M1 layer 5 cell
10uM Quinpirole IV -250pA to +400pA +50pA steps
+150pA

## Slide 18
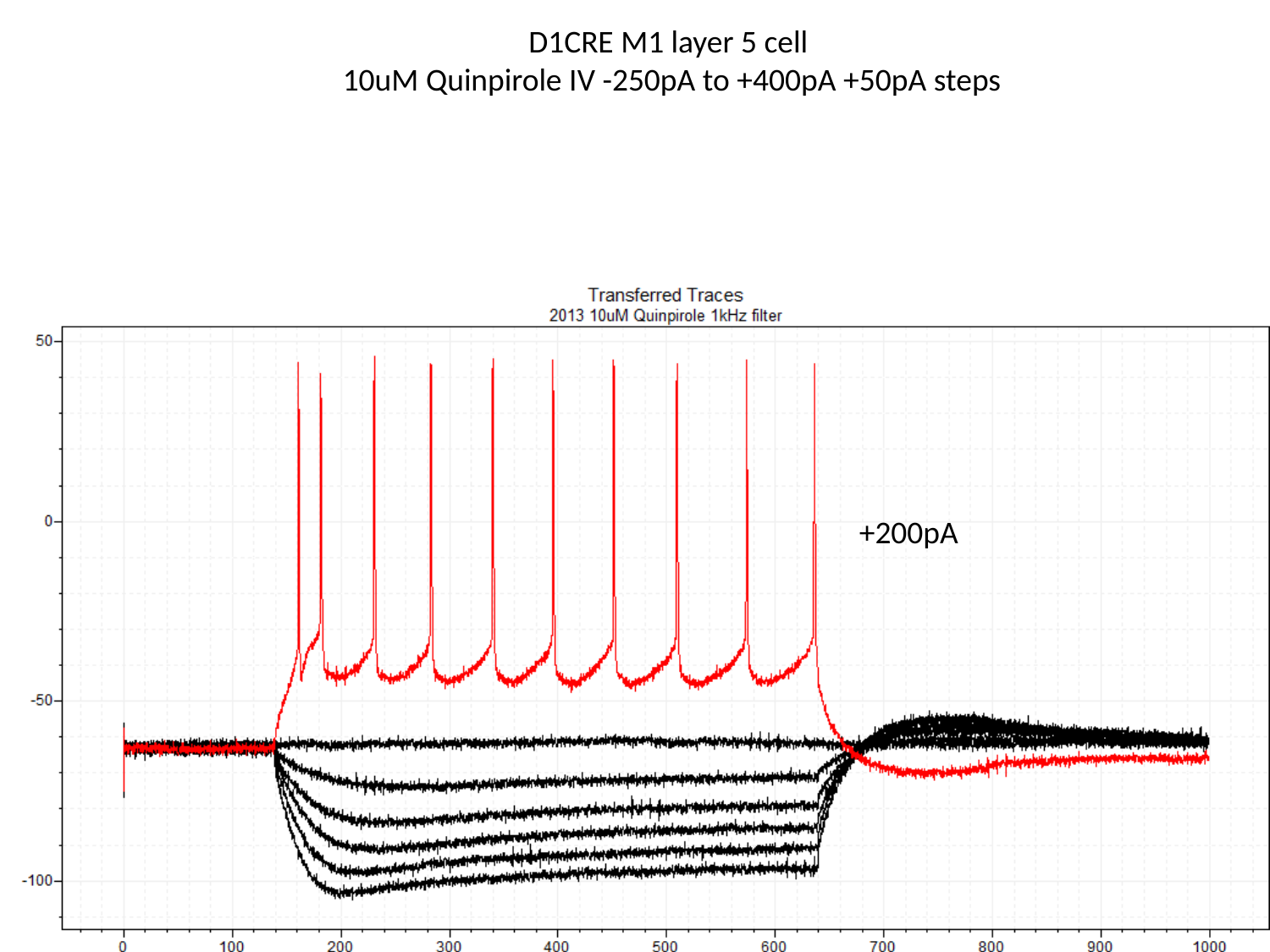

D1CRE M1 layer 5 cell
10uM Quinpirole IV -250pA to +400pA +50pA steps
+200pA

## Slide 19
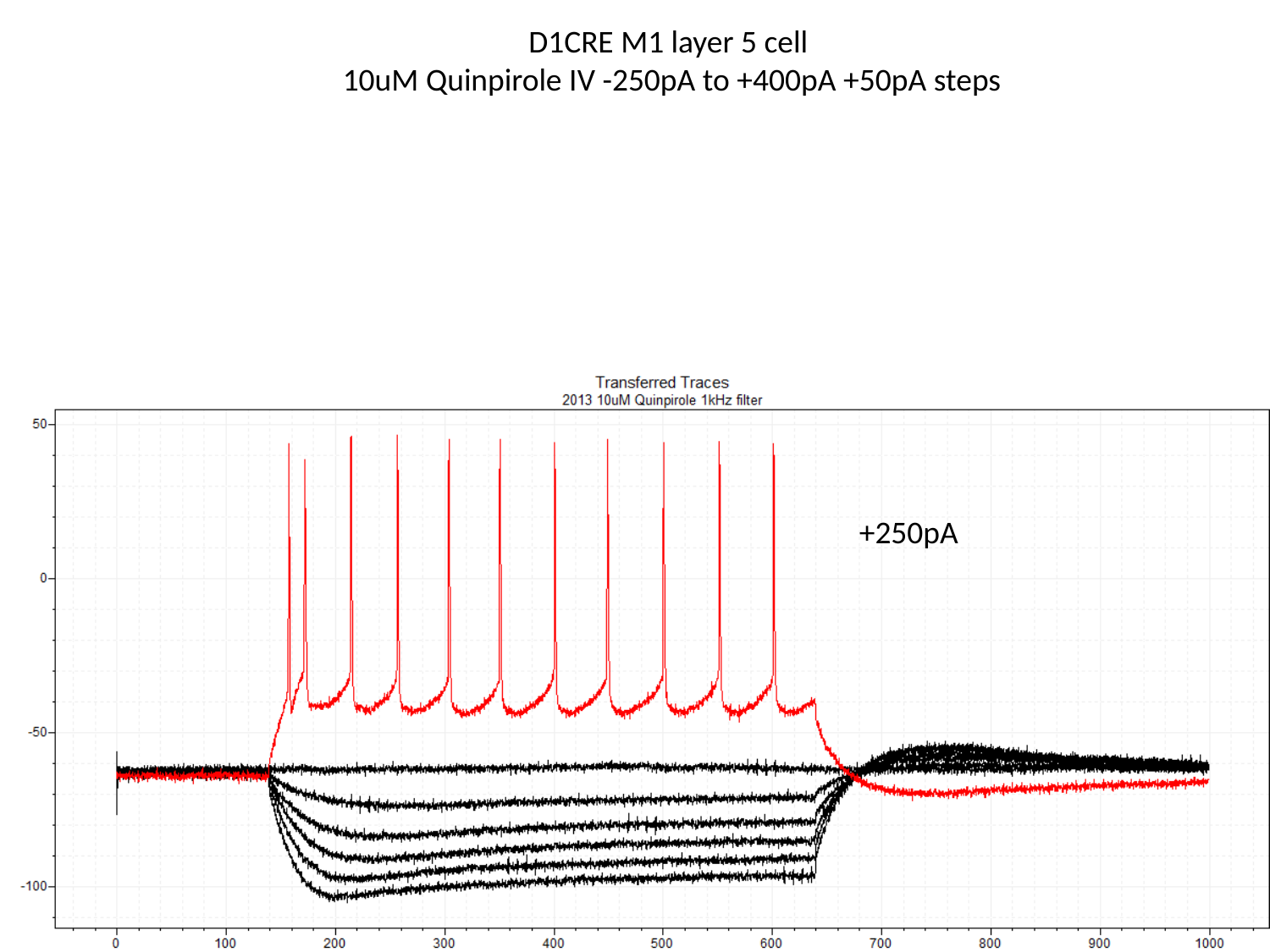

D1CRE M1 layer 5 cell
10uM Quinpirole IV -250pA to +400pA +50pA steps
+250pA

## Slide 20
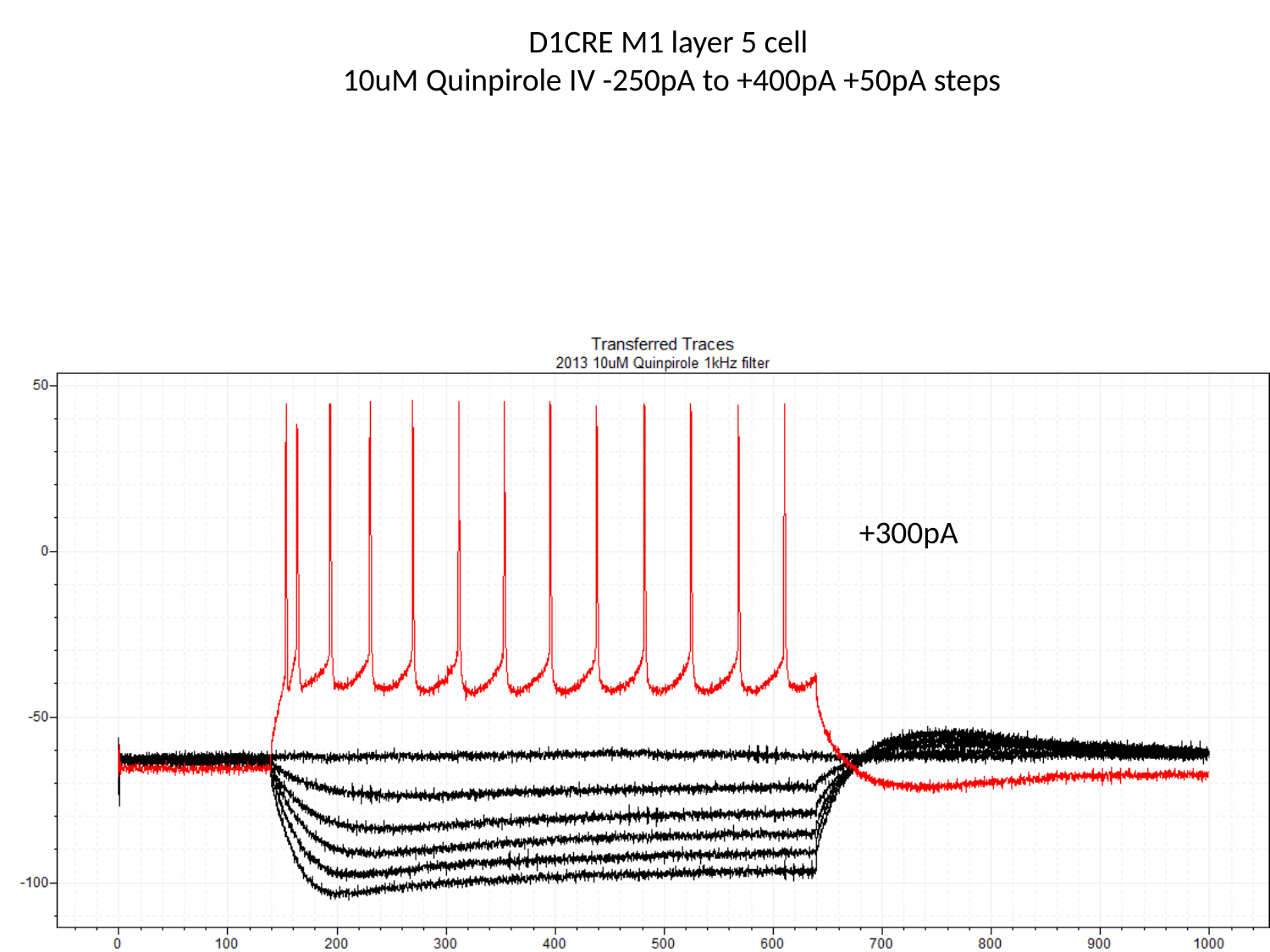

D1CRE M1 layer 5 cell
10uM Quinpirole IV -250pA to +400pA +50pA steps
+300pA

## Slide 21
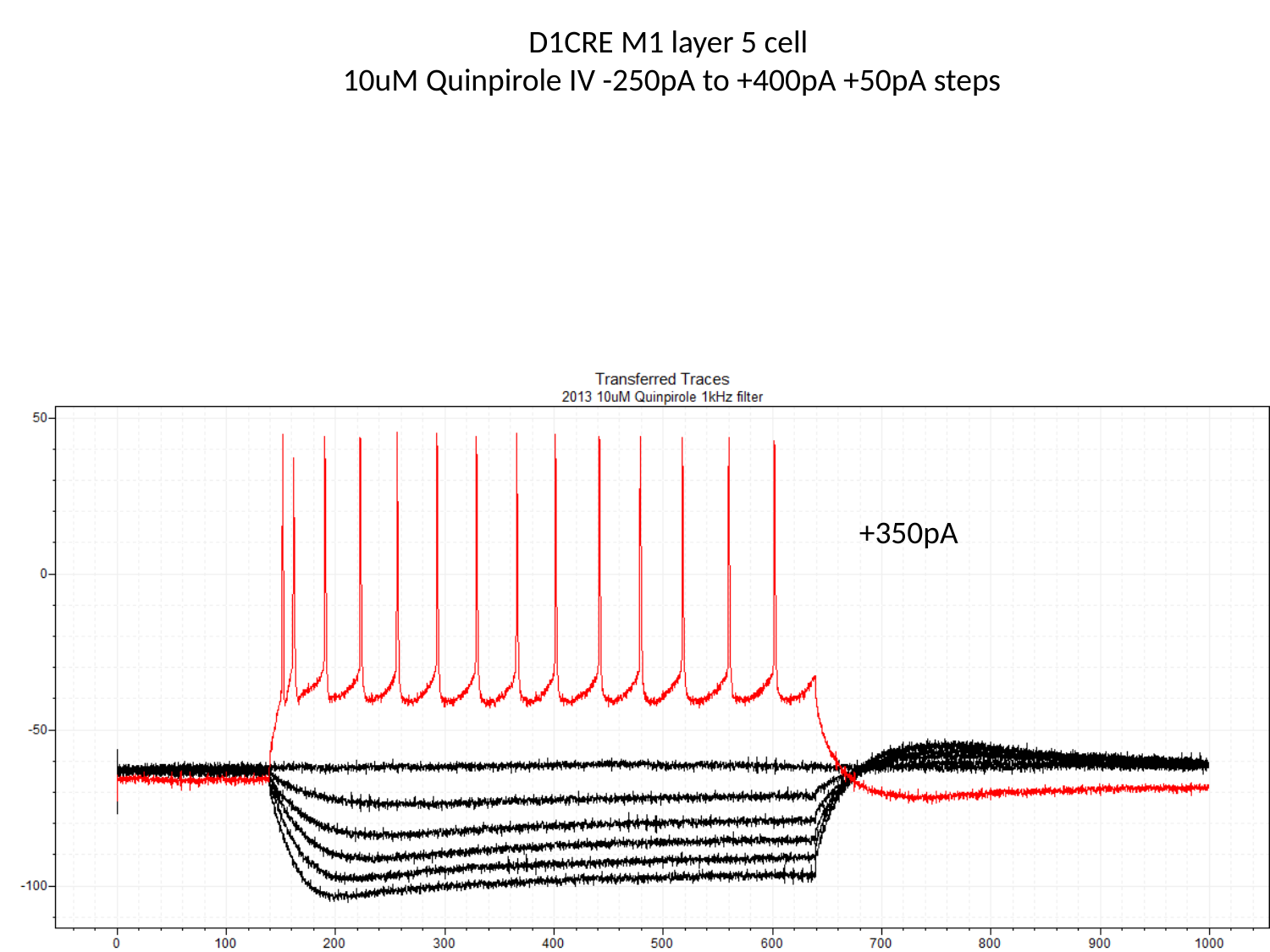

D1CRE M1 layer 5 cell
10uM Quinpirole IV -250pA to +400pA +50pA steps
+350pA

## Slide 22
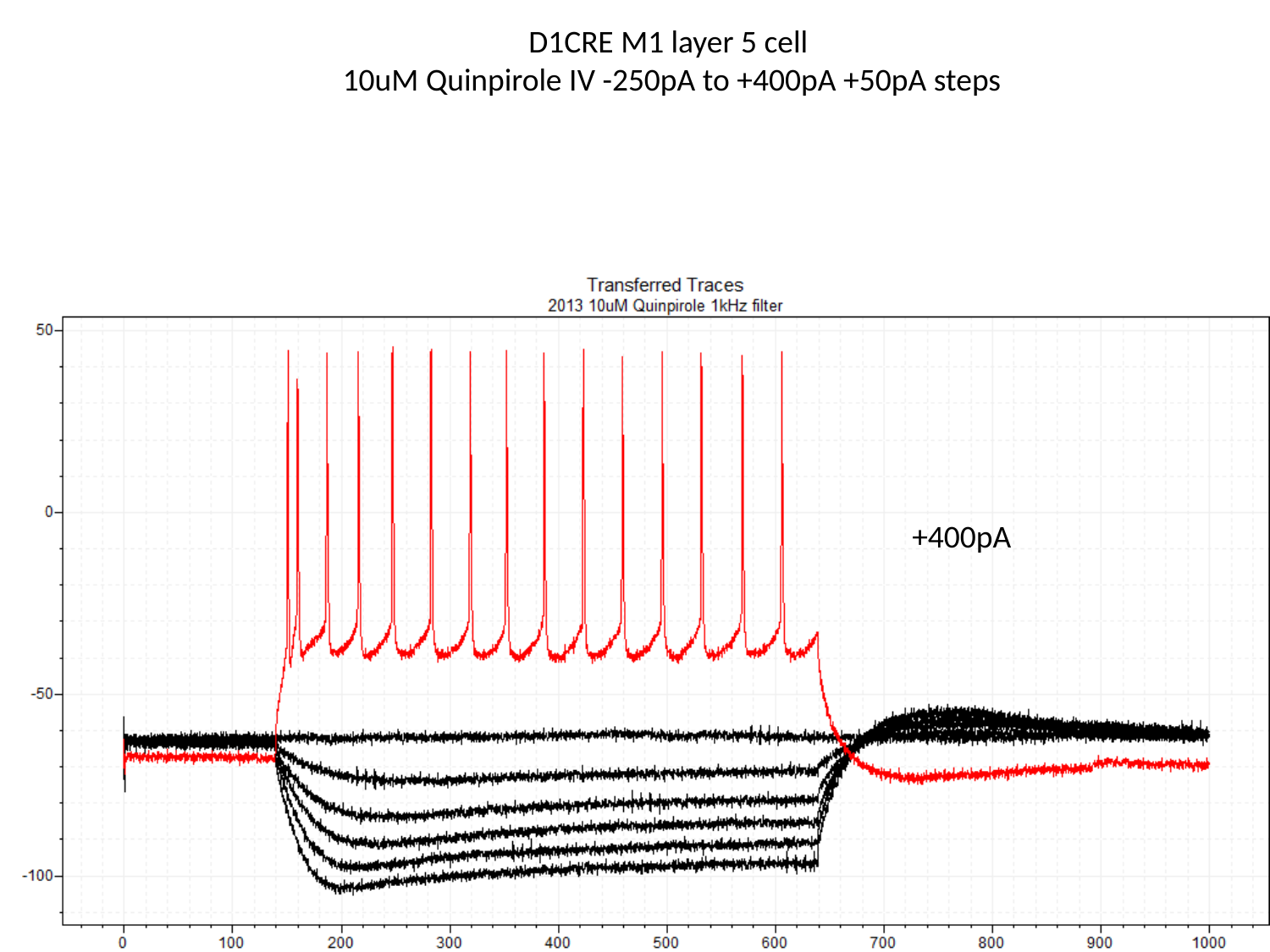

D1CRE M1 layer 5 cell
10uM Quinpirole IV -250pA to +400pA +50pA steps
+400pA

## Slide 23
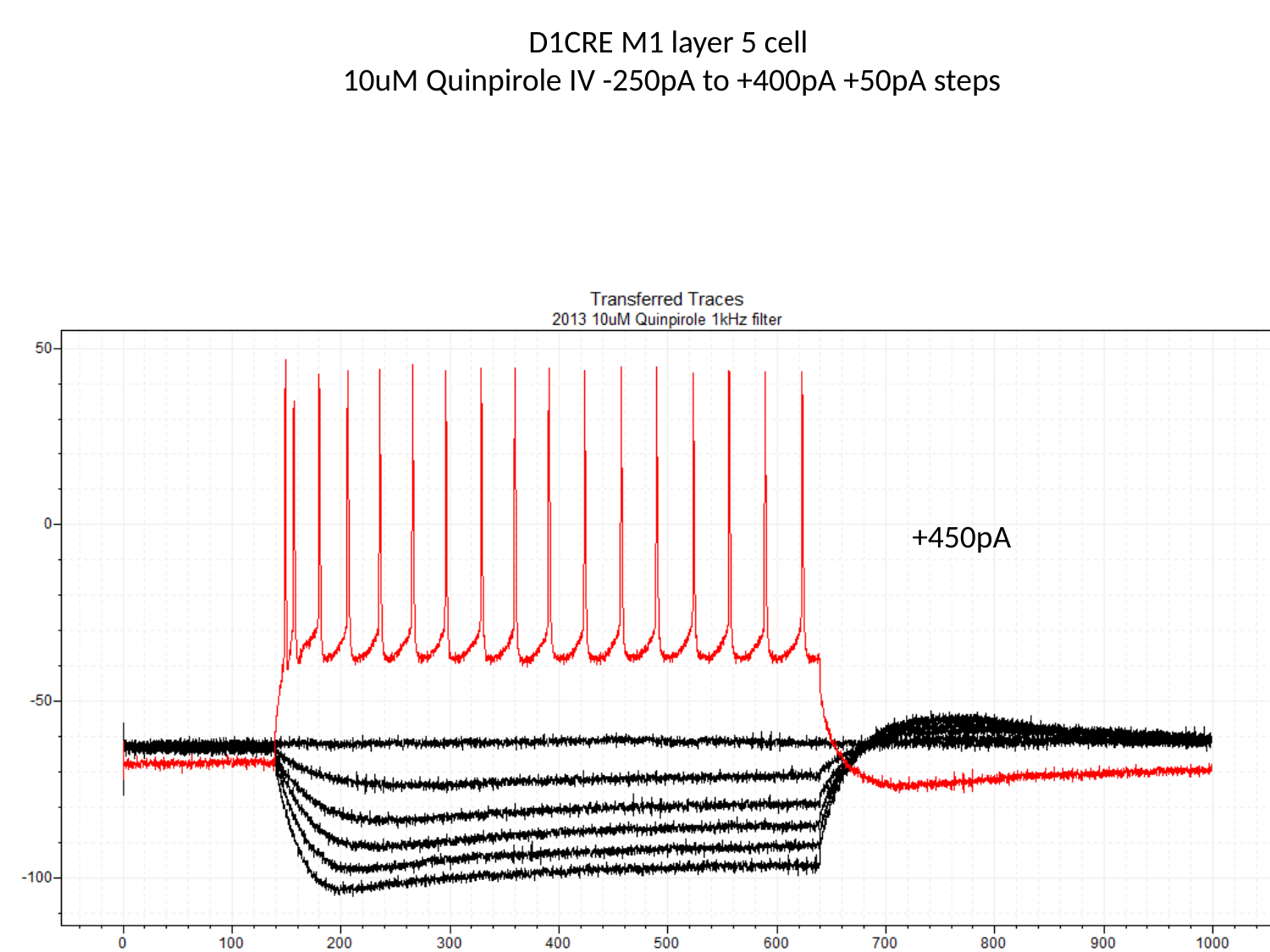

D1CRE M1 layer 5 cell
10uM Quinpirole IV -250pA to +400pA +50pA steps
+450pA

## Slide 24
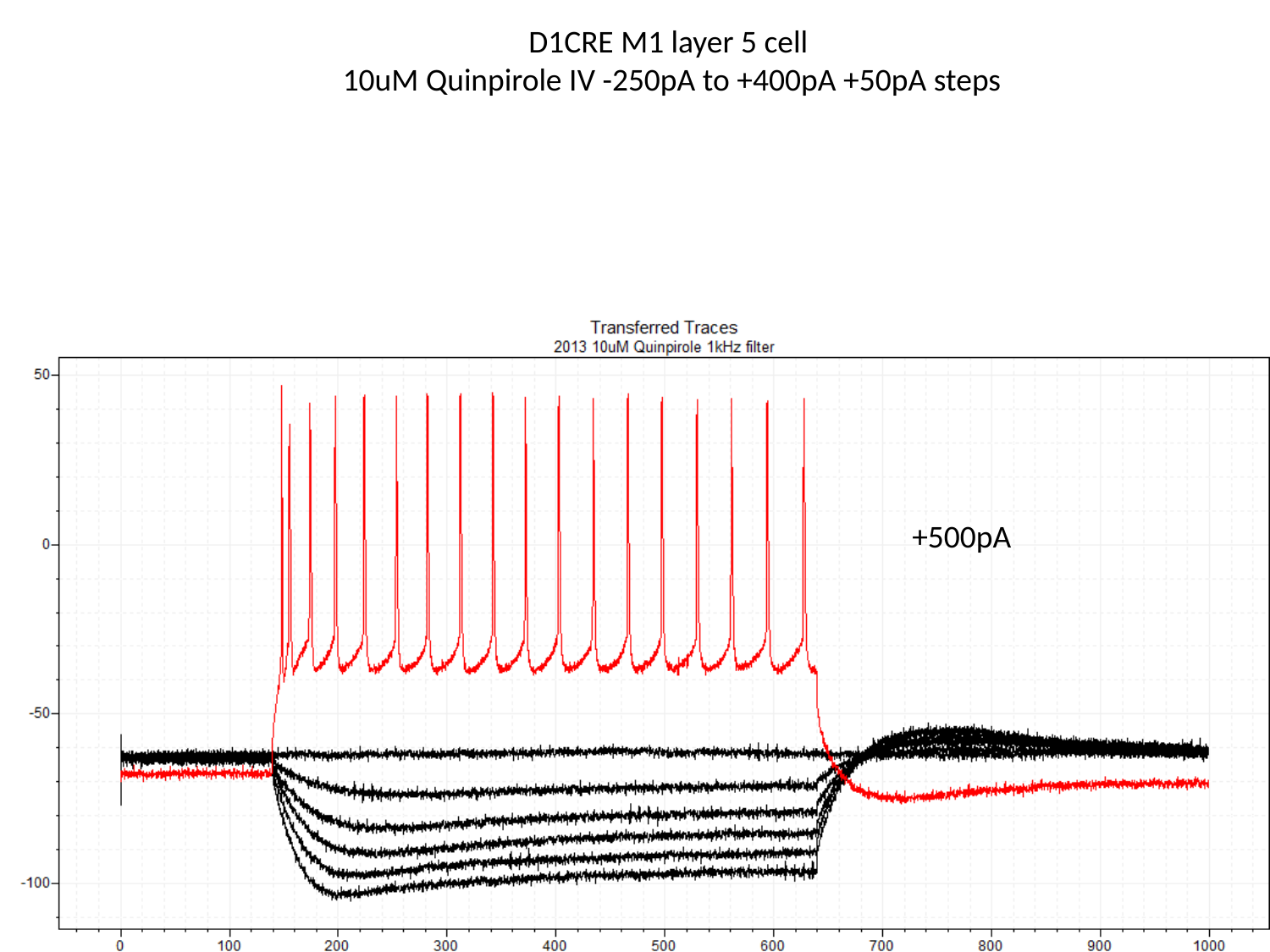

D1CRE M1 layer 5 cell
10uM Quinpirole IV -250pA to +400pA +50pA steps
+500pA

## Slide 25
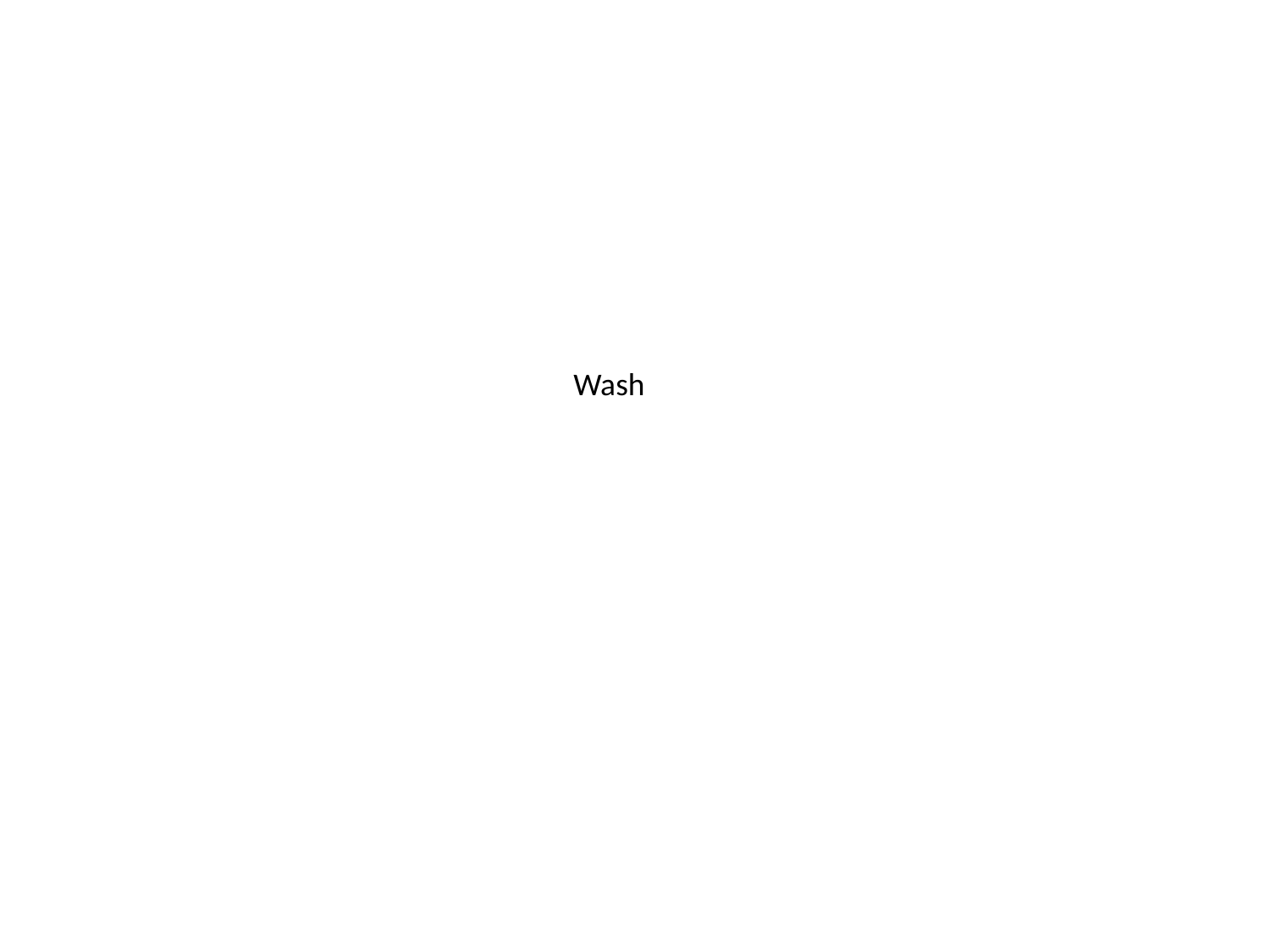

Wash

## Slide 26
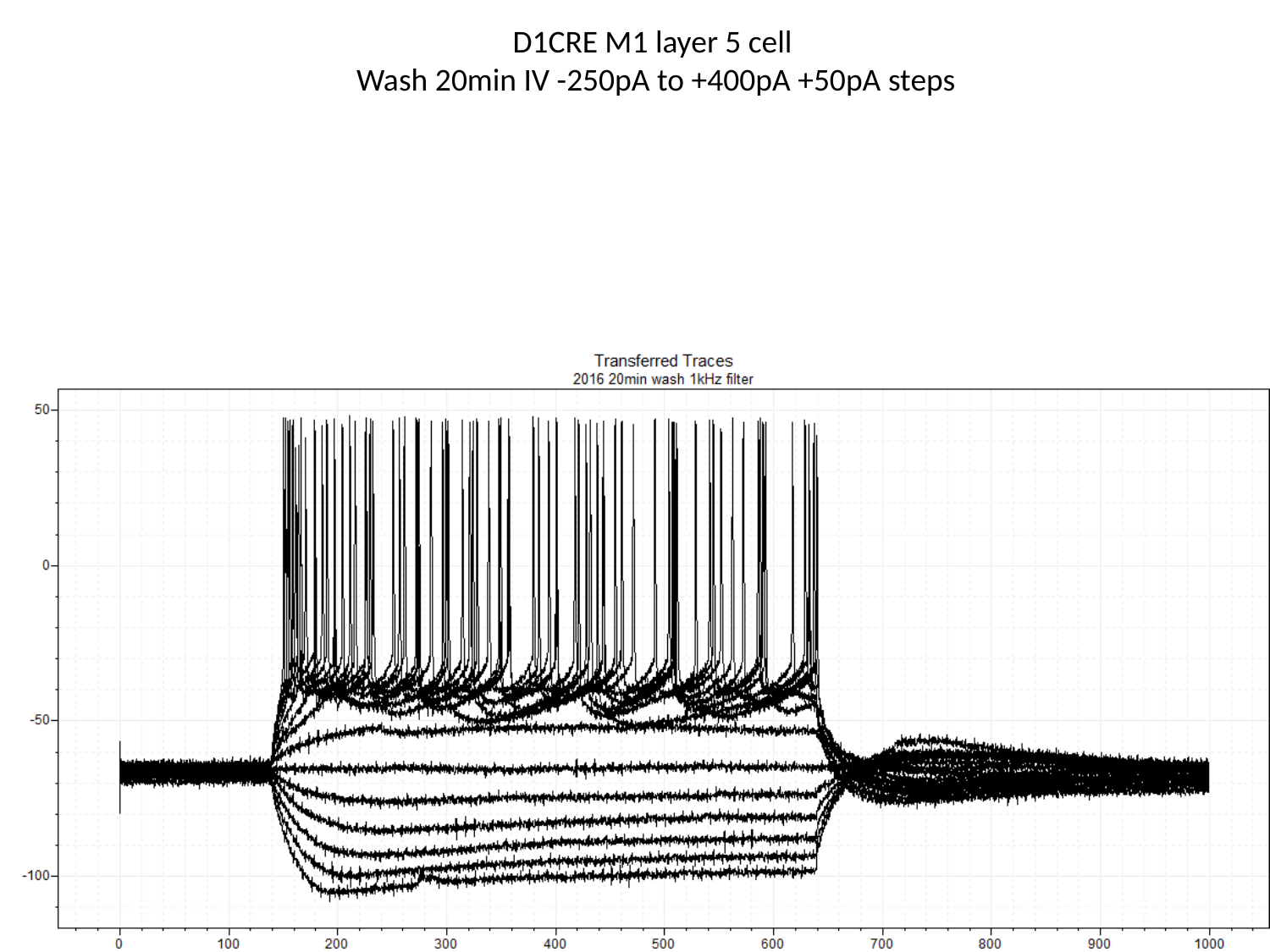

D1CRE M1 layer 5 cell
Wash 20min IV -250pA to +400pA +50pA steps

## Slide 27
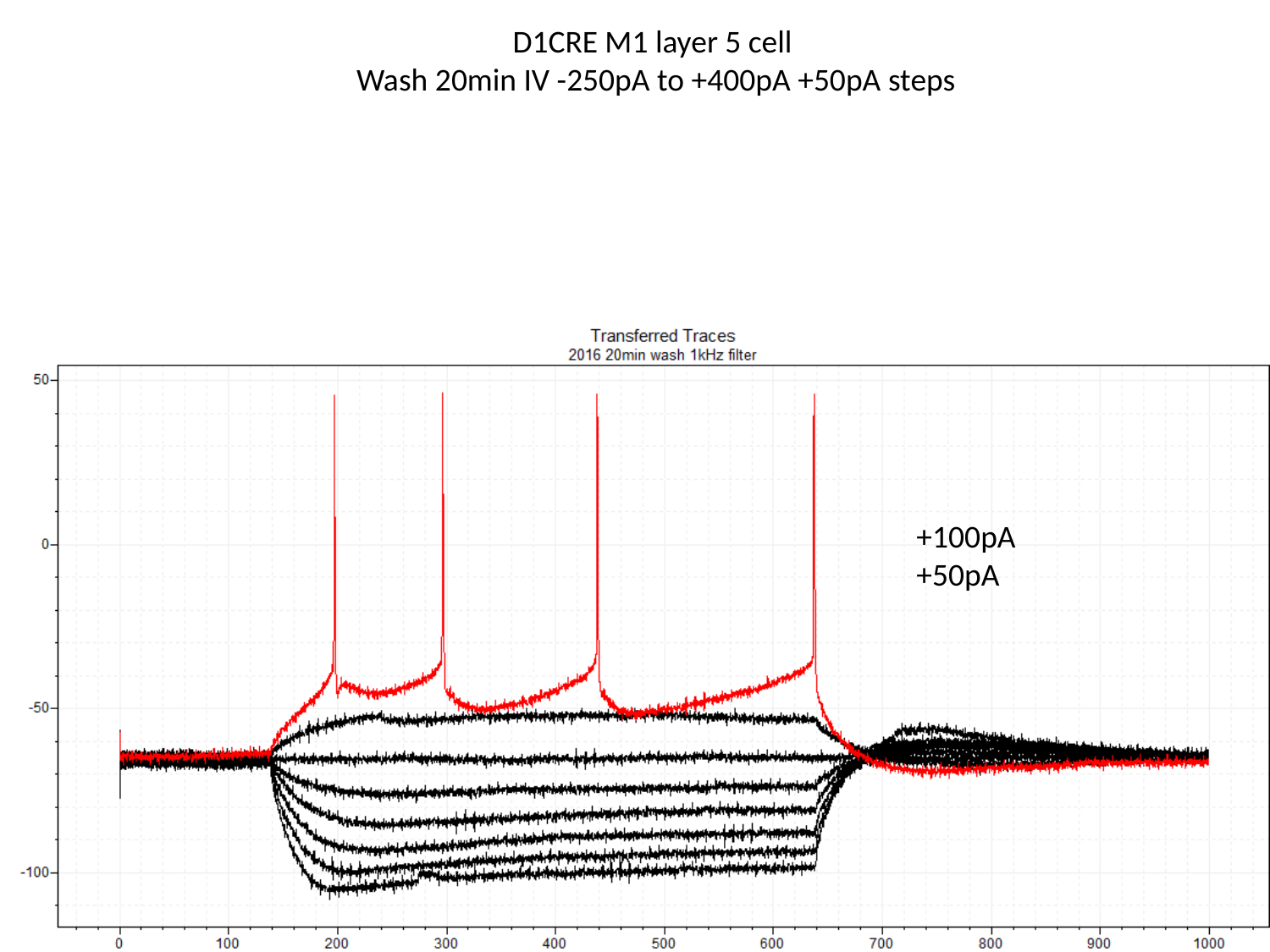

D1CRE M1 layer 5 cell
Wash 20min IV -250pA to +400pA +50pA steps
+100pA
+50pA

## Slide 28
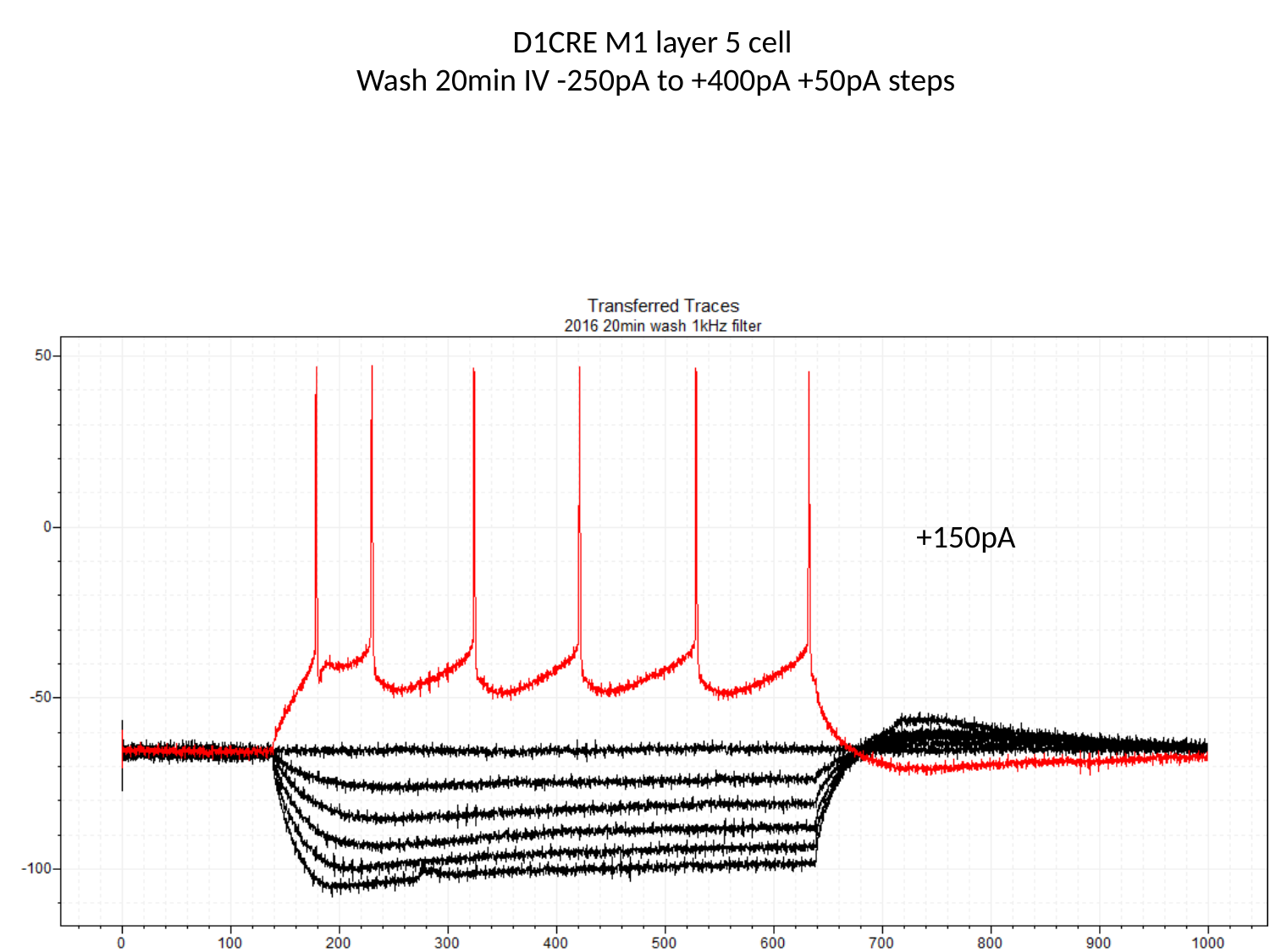

D1CRE M1 layer 5 cell
Wash 20min IV -250pA to +400pA +50pA steps
+150pA

## Slide 29
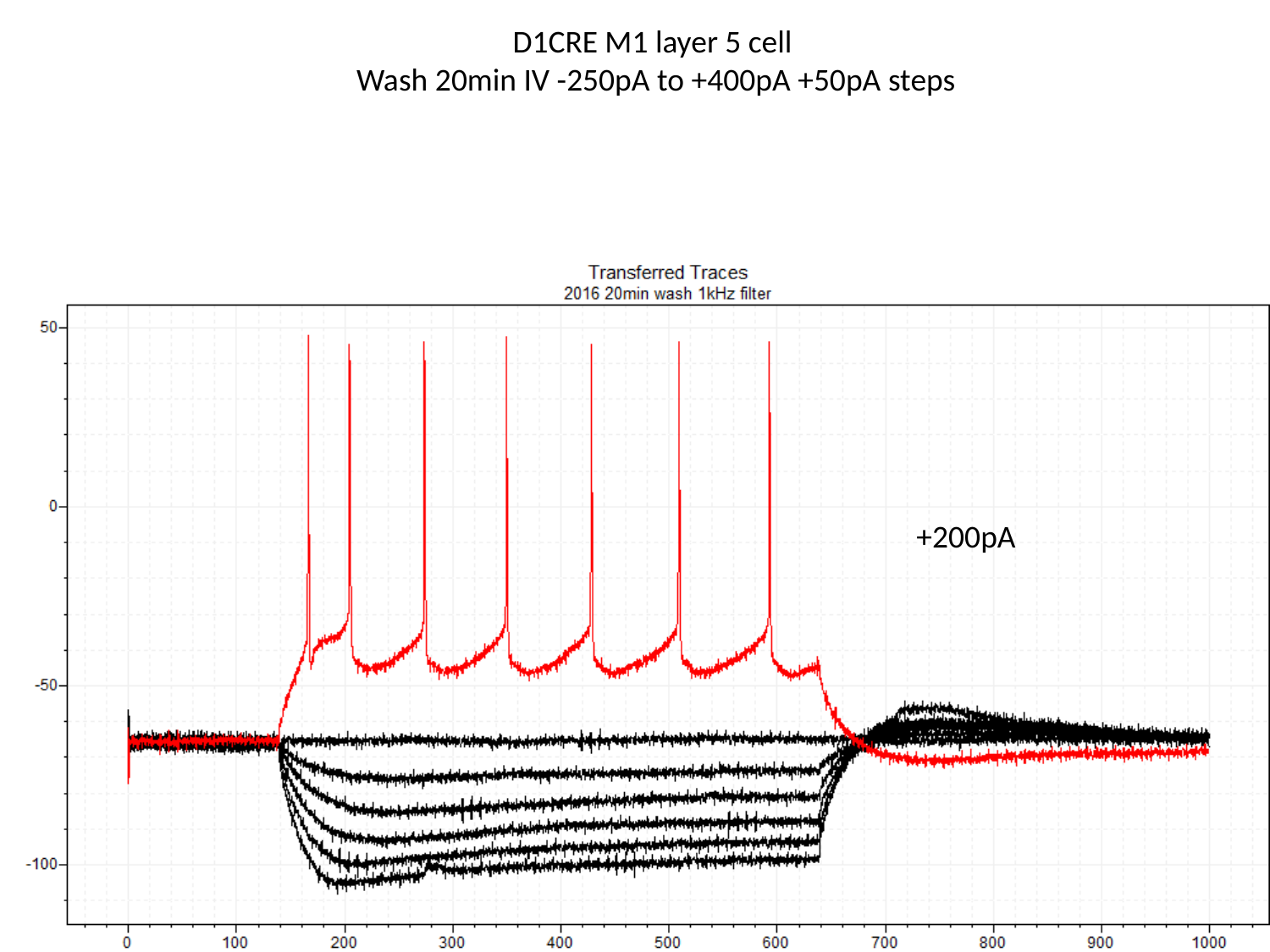

D1CRE M1 layer 5 cell
Wash 20min IV -250pA to +400pA +50pA steps
+200pA

## Slide 30
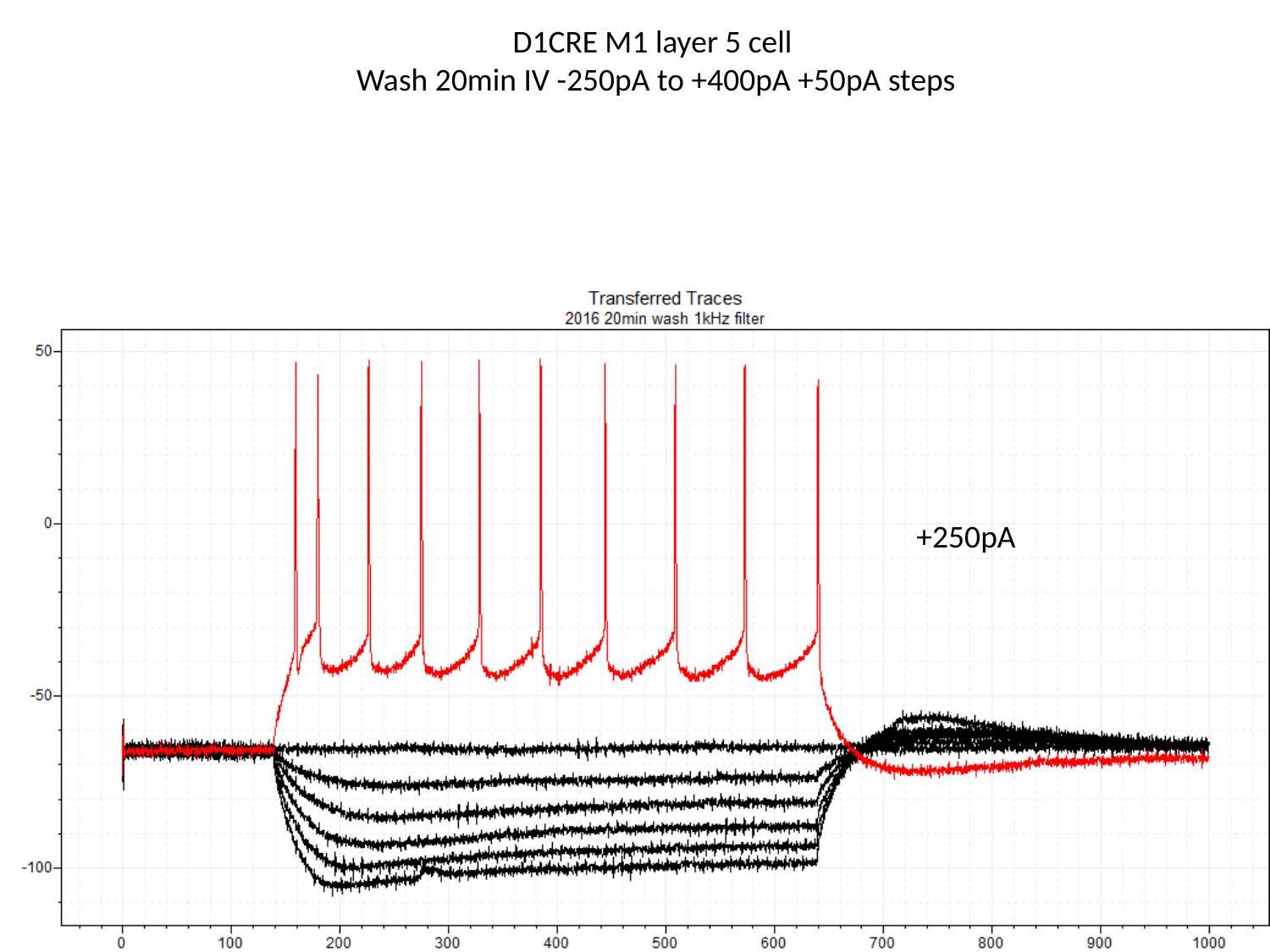

D1CRE M1 layer 5 cell
Wash 20min IV -250pA to +400pA +50pA steps
+250pA

## Slide 31
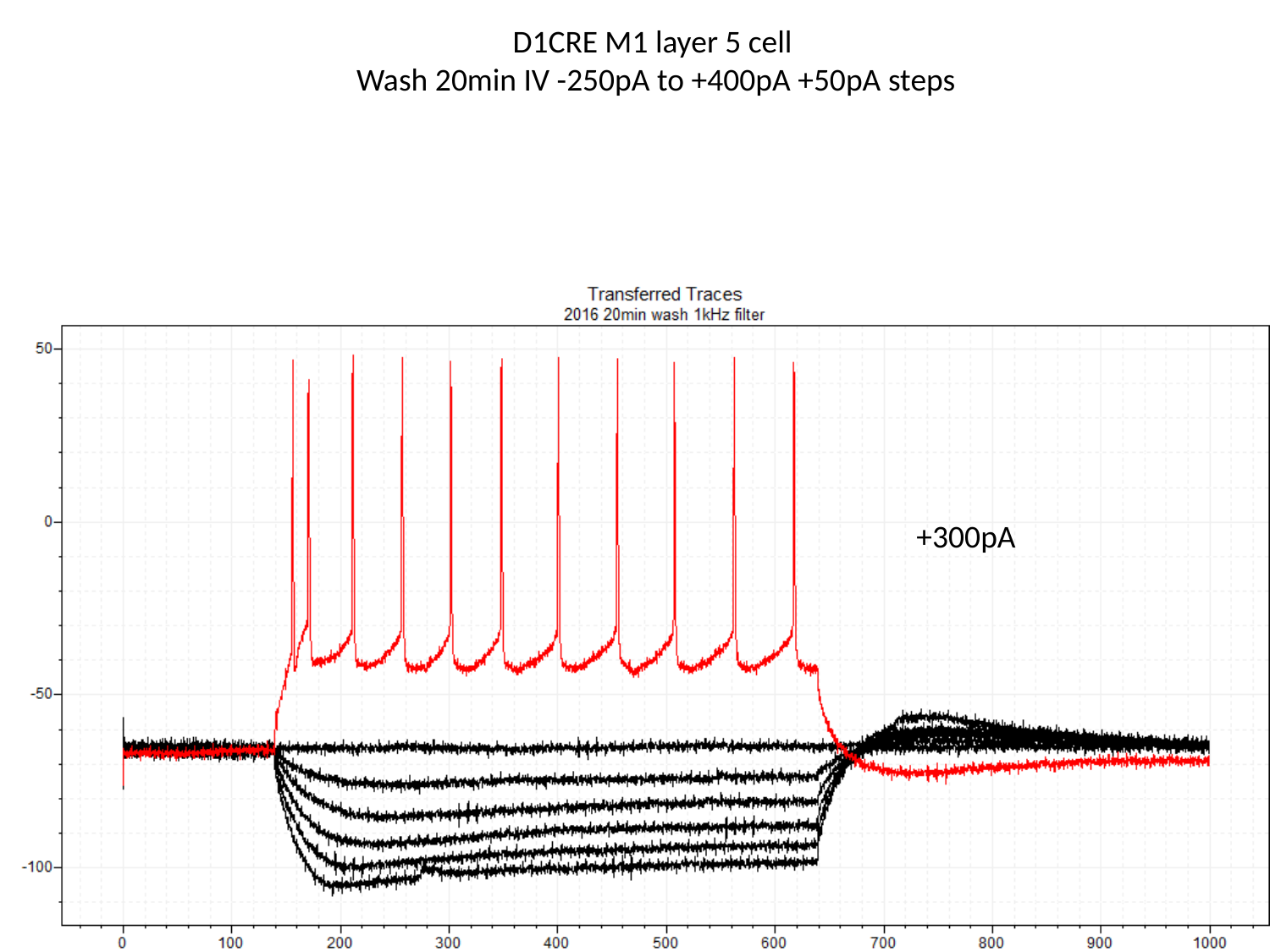

D1CRE M1 layer 5 cell
Wash 20min IV -250pA to +400pA +50pA steps
+300pA

## Slide 32
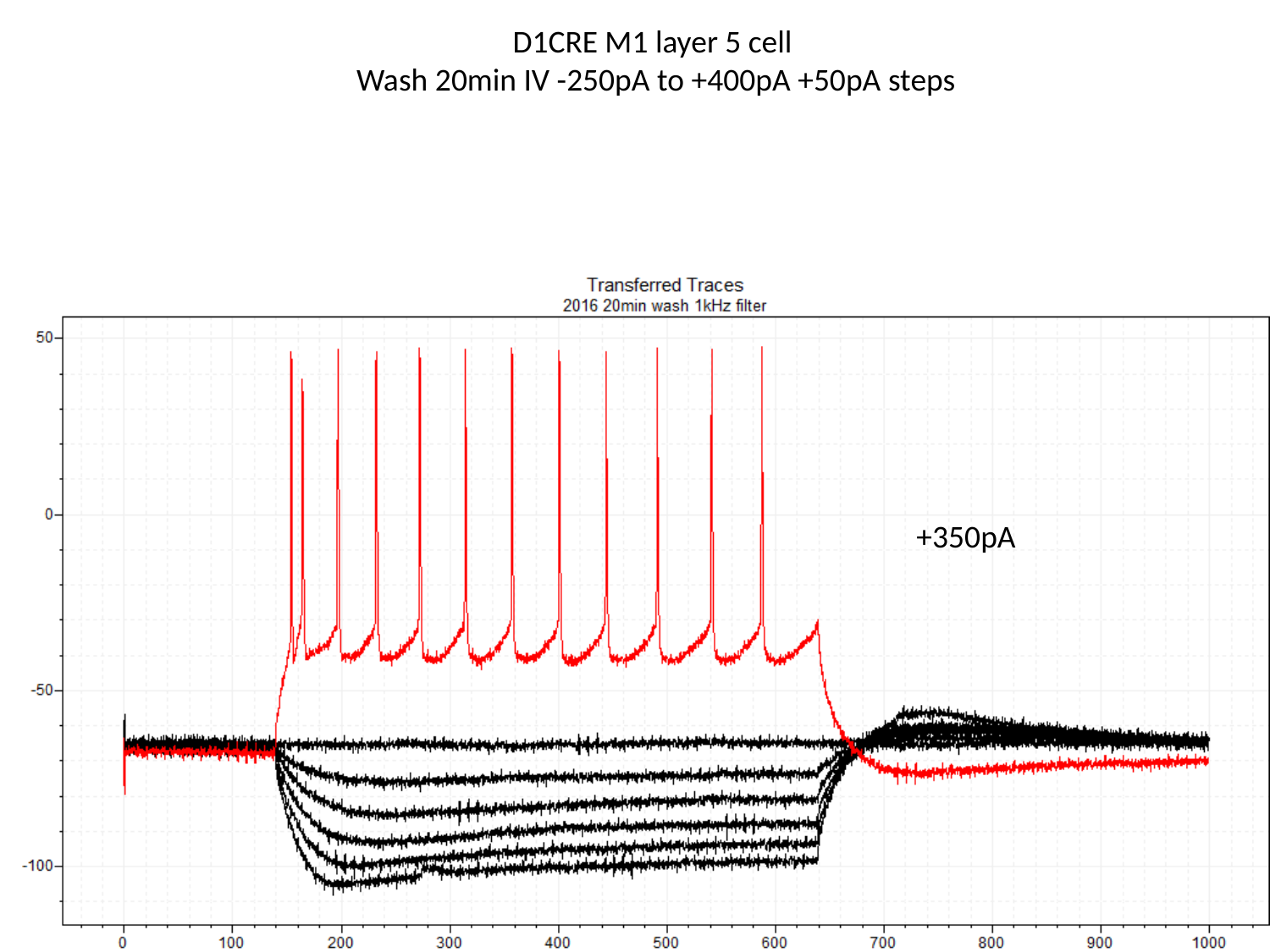

D1CRE M1 layer 5 cell
Wash 20min IV -250pA to +400pA +50pA steps
+350pA

## Slide 33
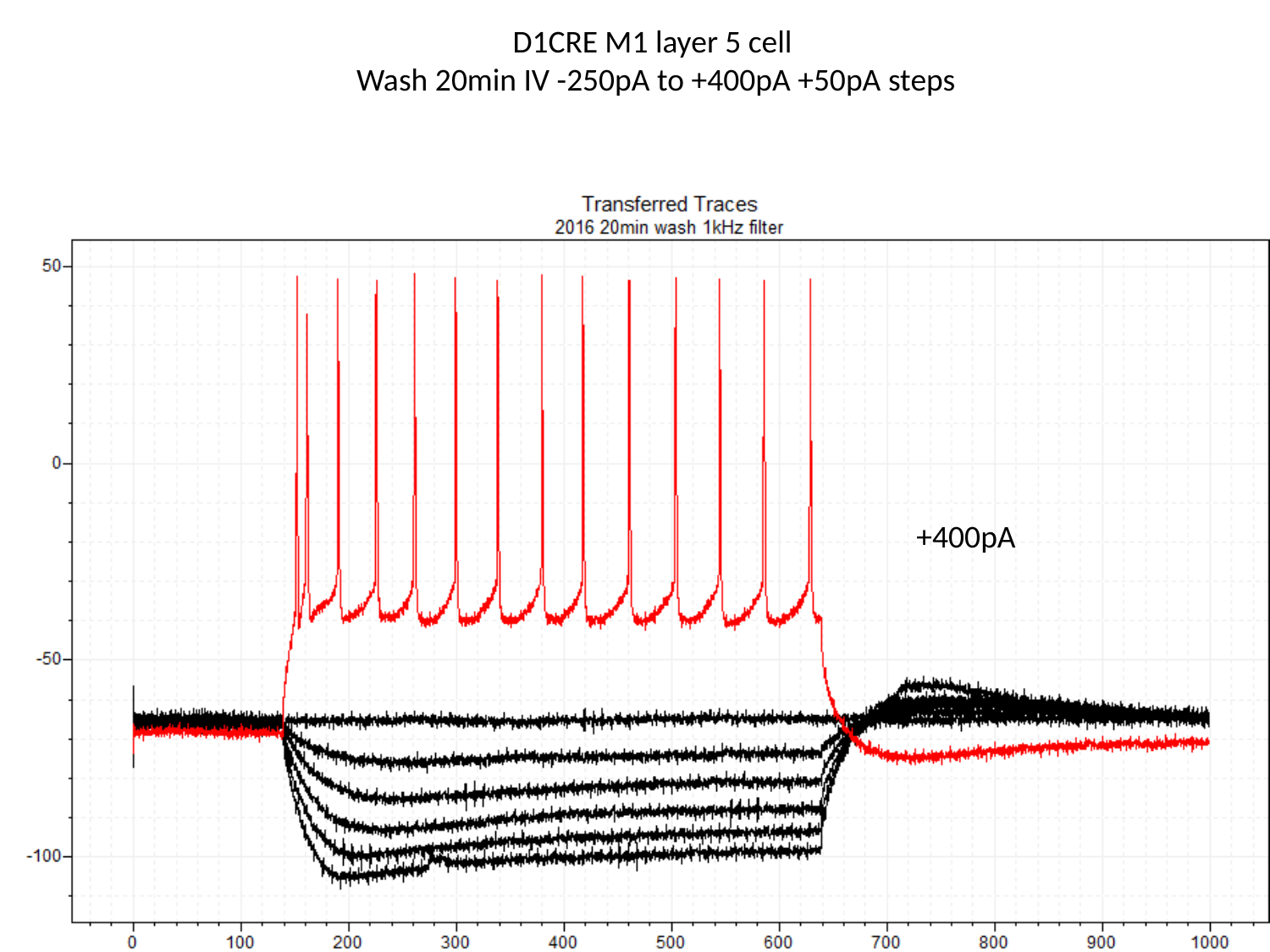

D1CRE M1 layer 5 cell
Wash 20min IV -250pA to +400pA +50pA steps
+400pA

## Slide 34
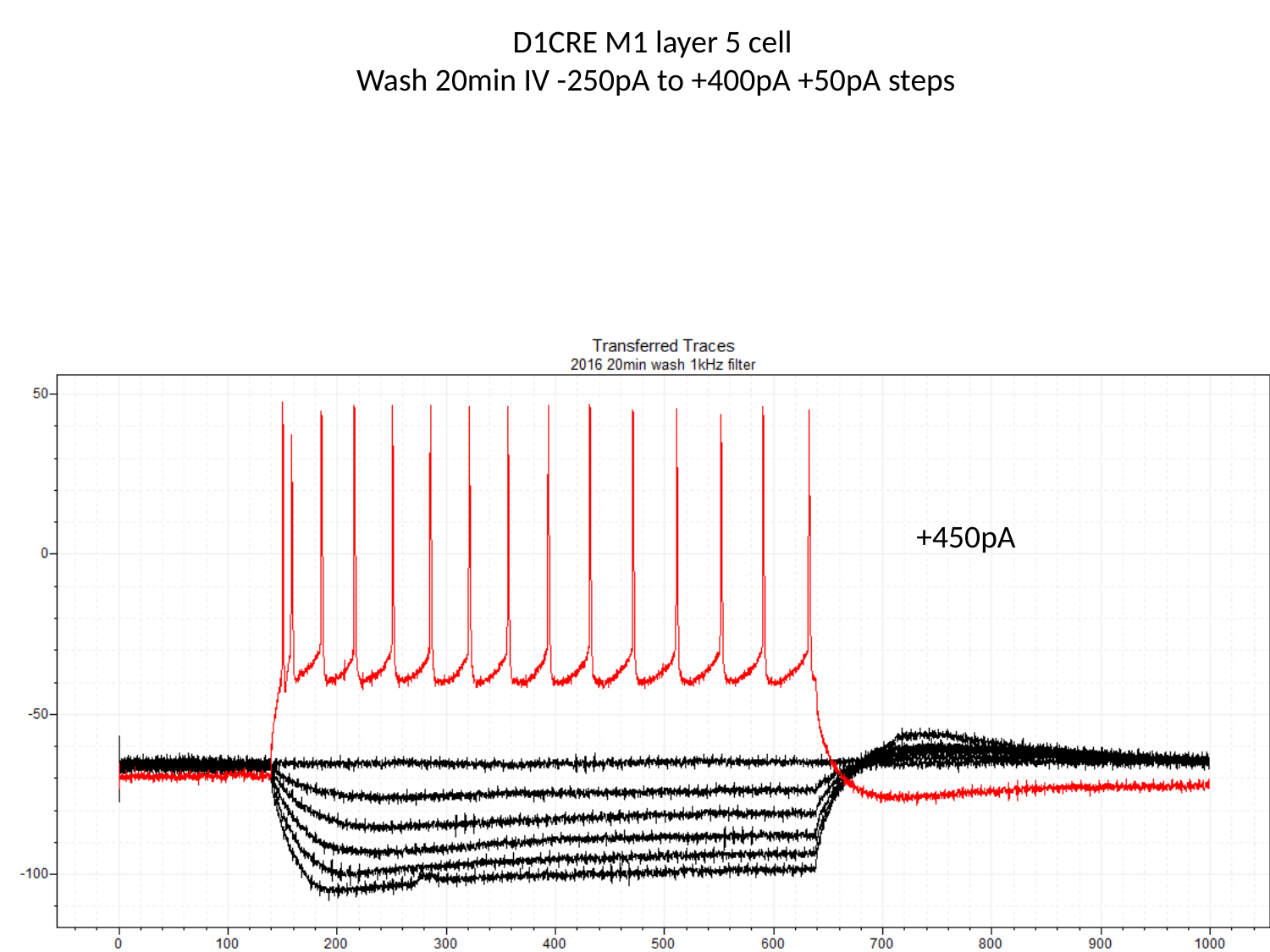

D1CRE M1 layer 5 cell
Wash 20min IV -250pA to +400pA +50pA steps
+450pA

## Slide 35
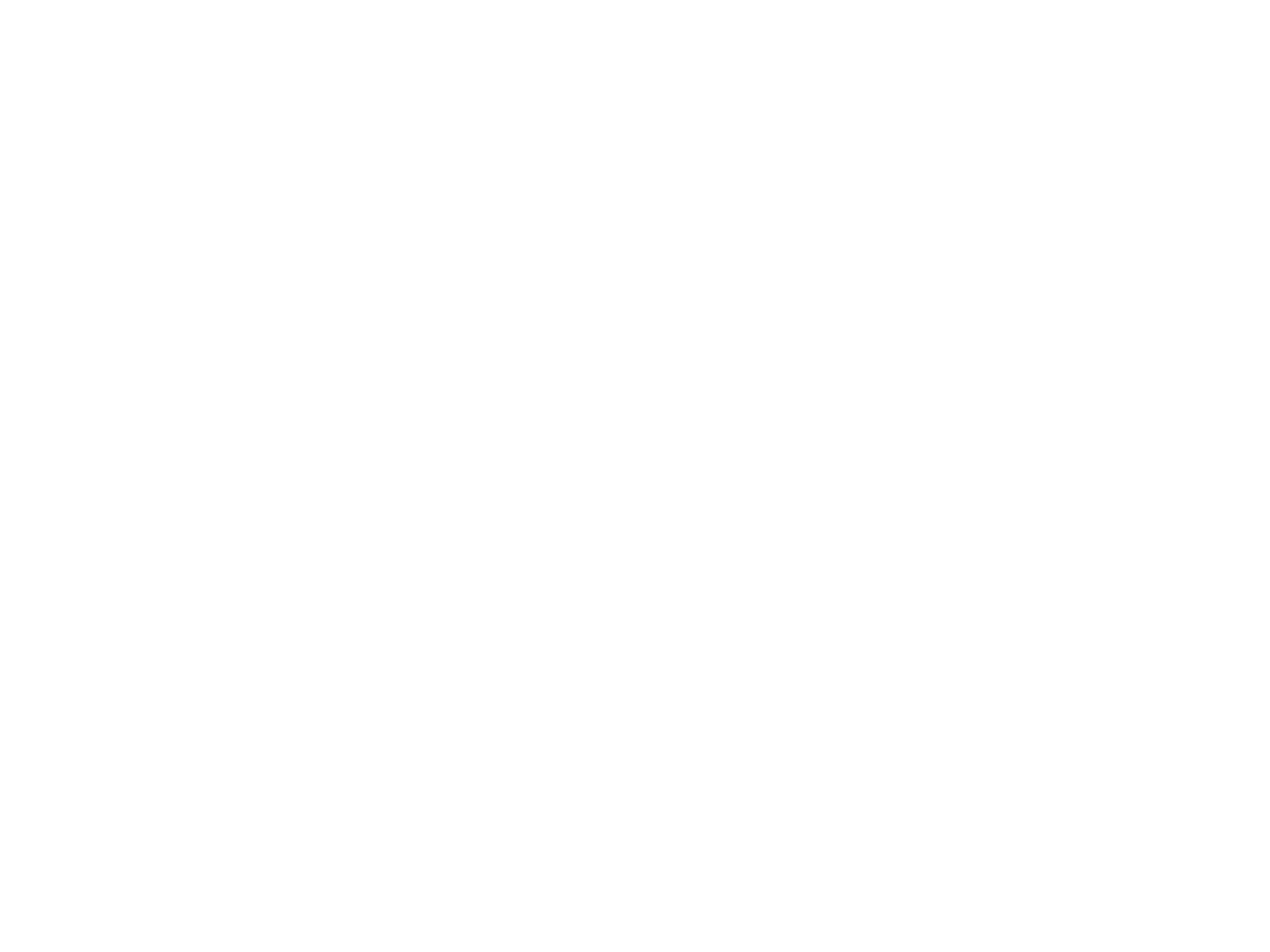

## Slide 36
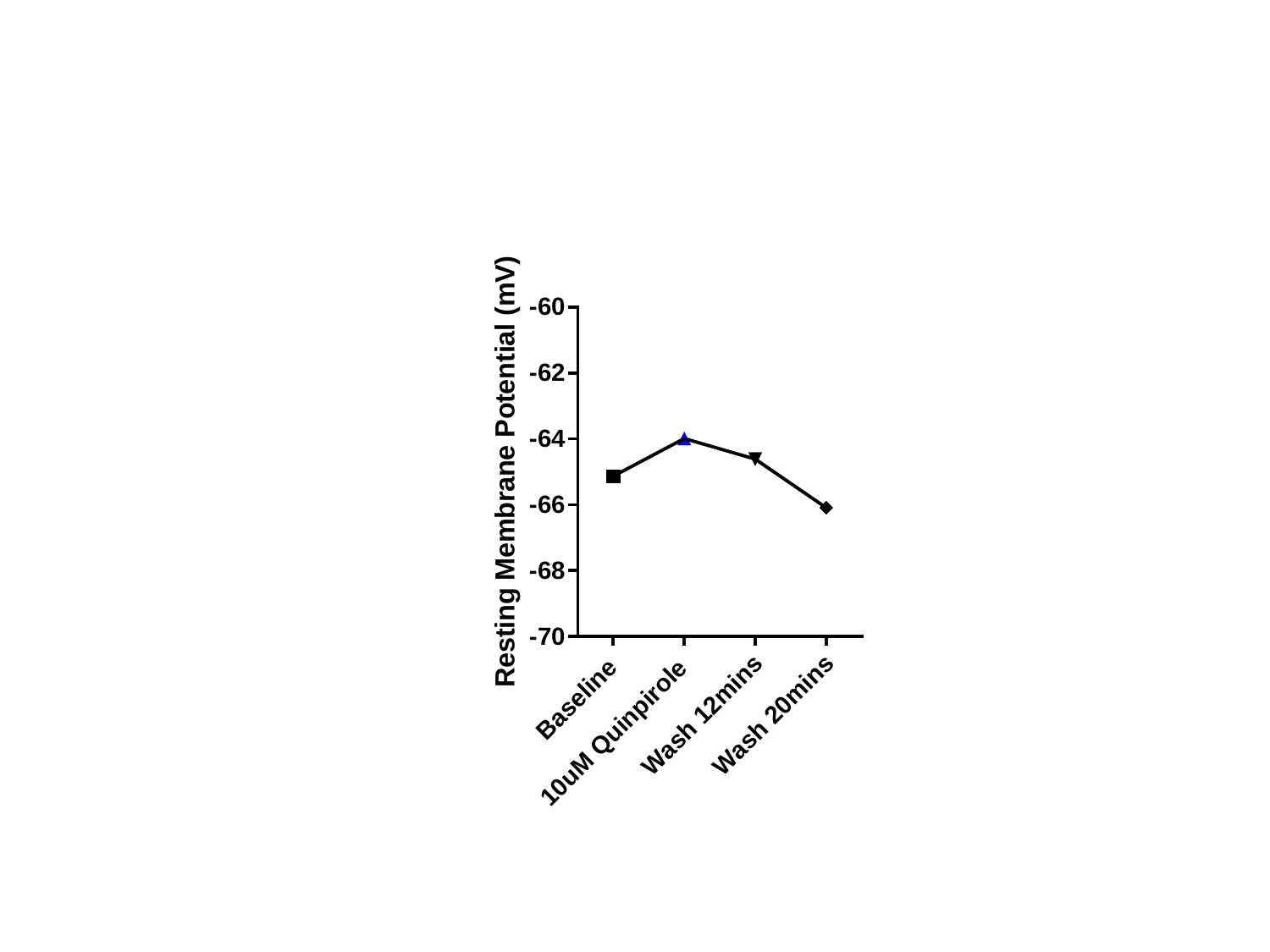

## Slide 37
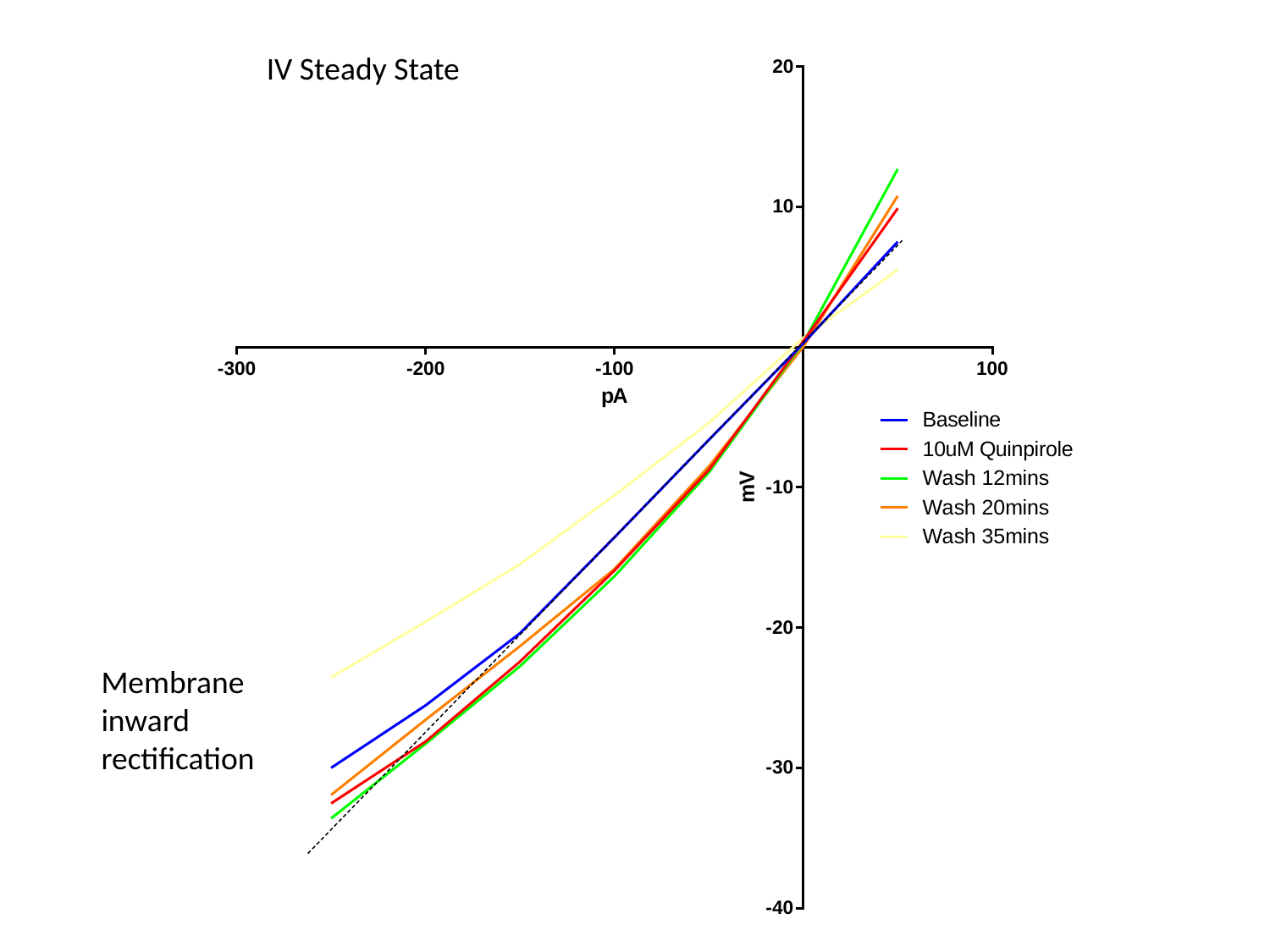

IV Steady State
Membrane inward rectification

## Slide 38
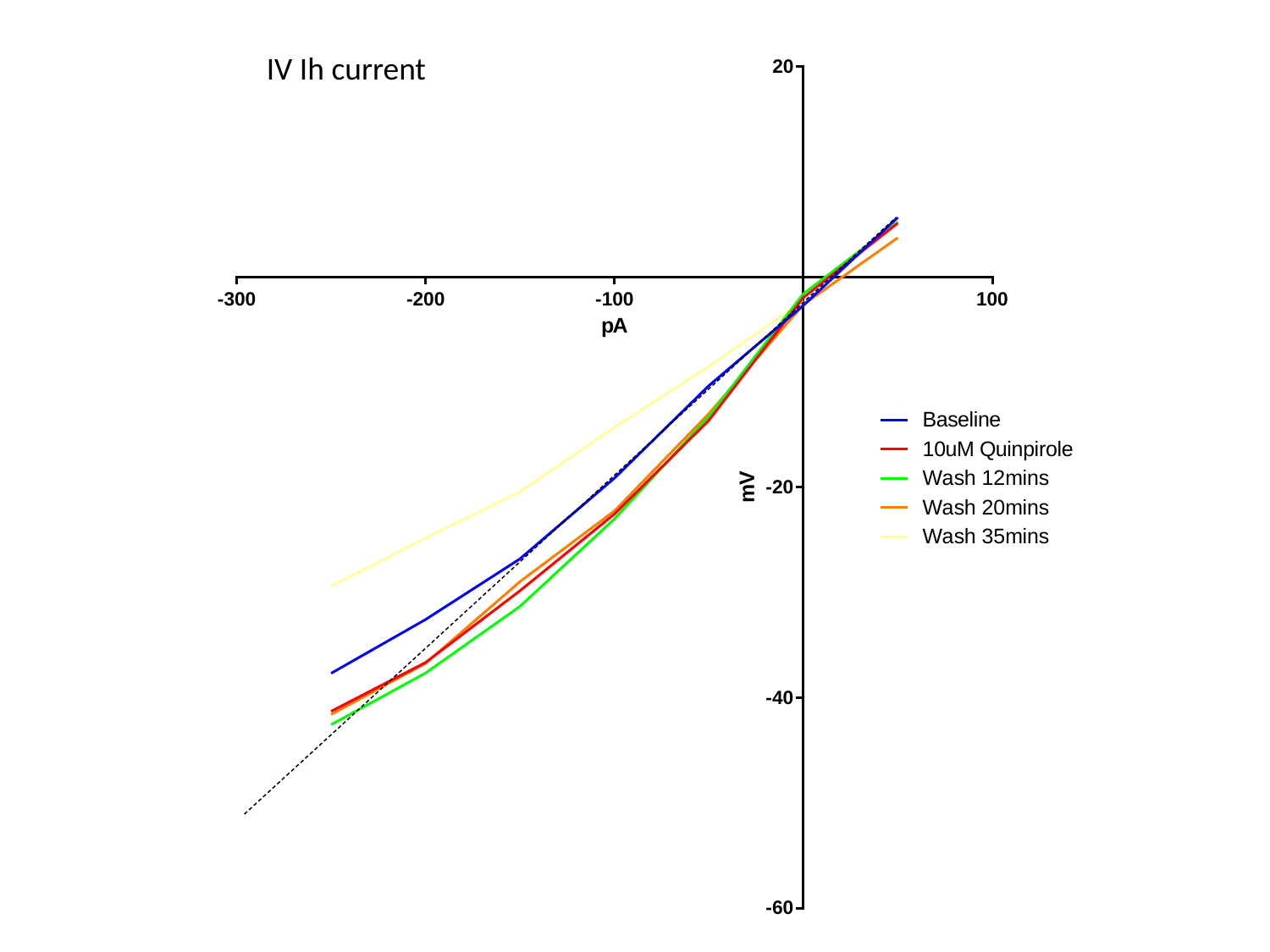

IV Ih current

## Slide 39
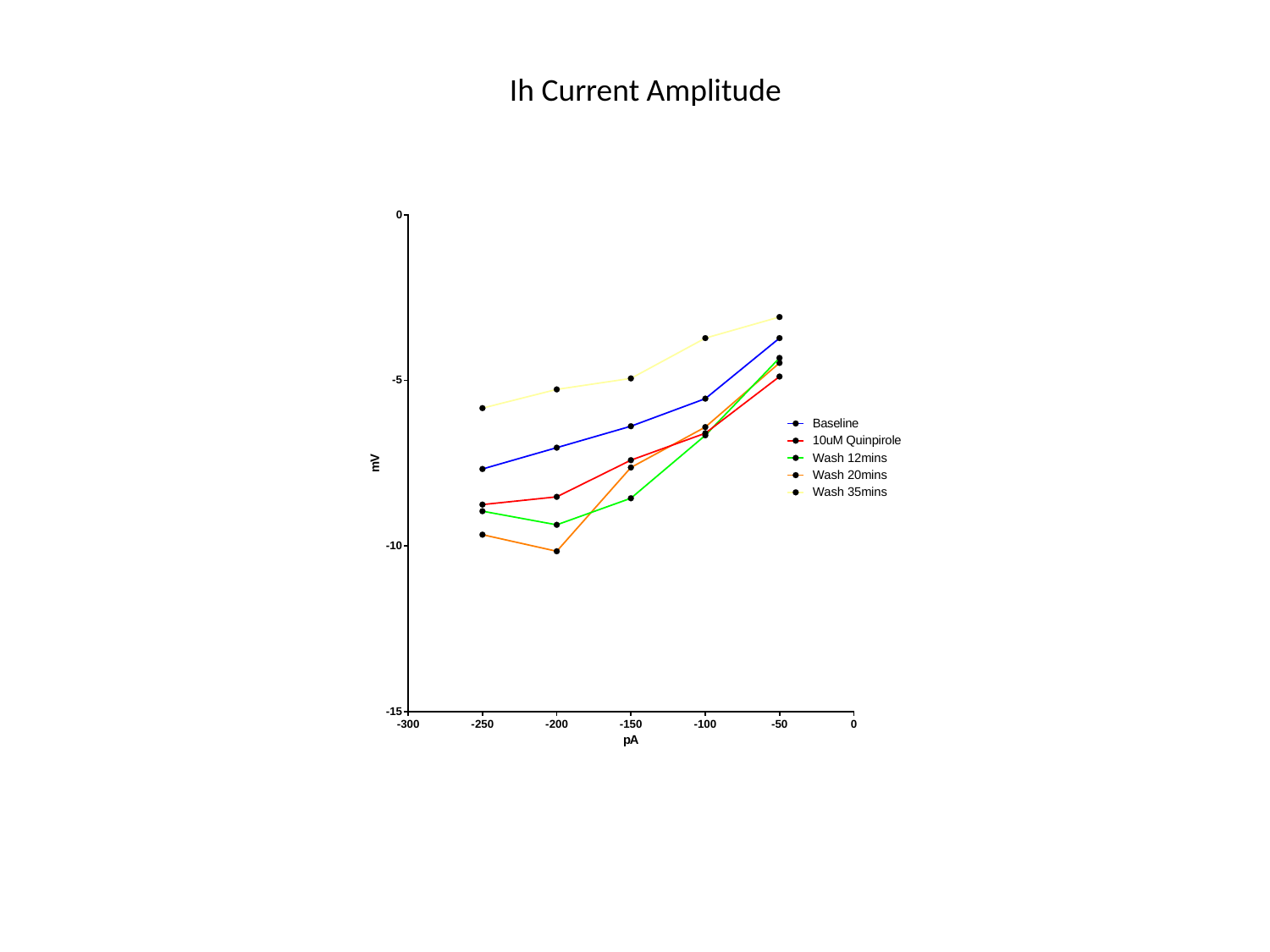

Ih Current Amplitude

## Slide 40
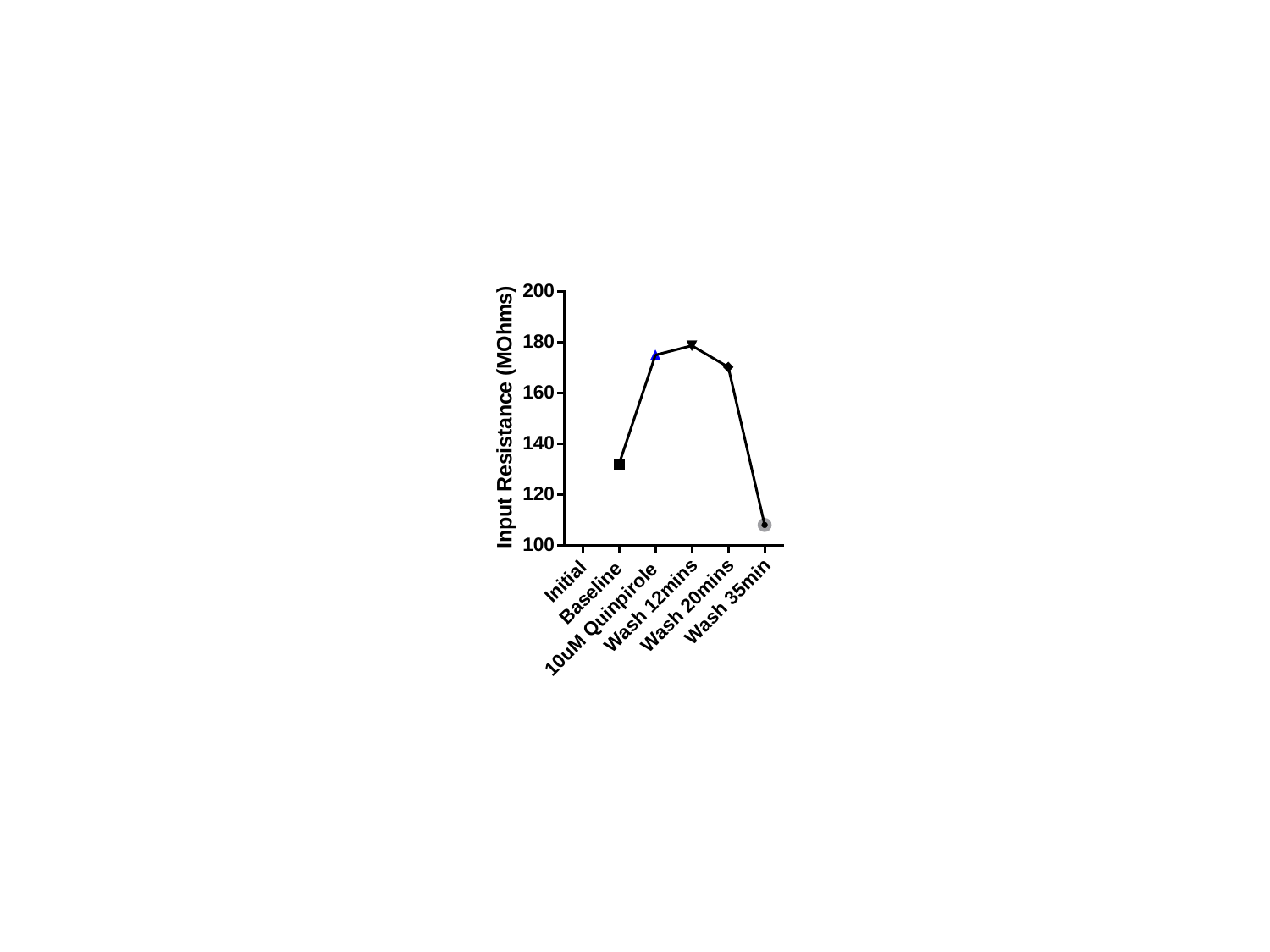

## Slide 41
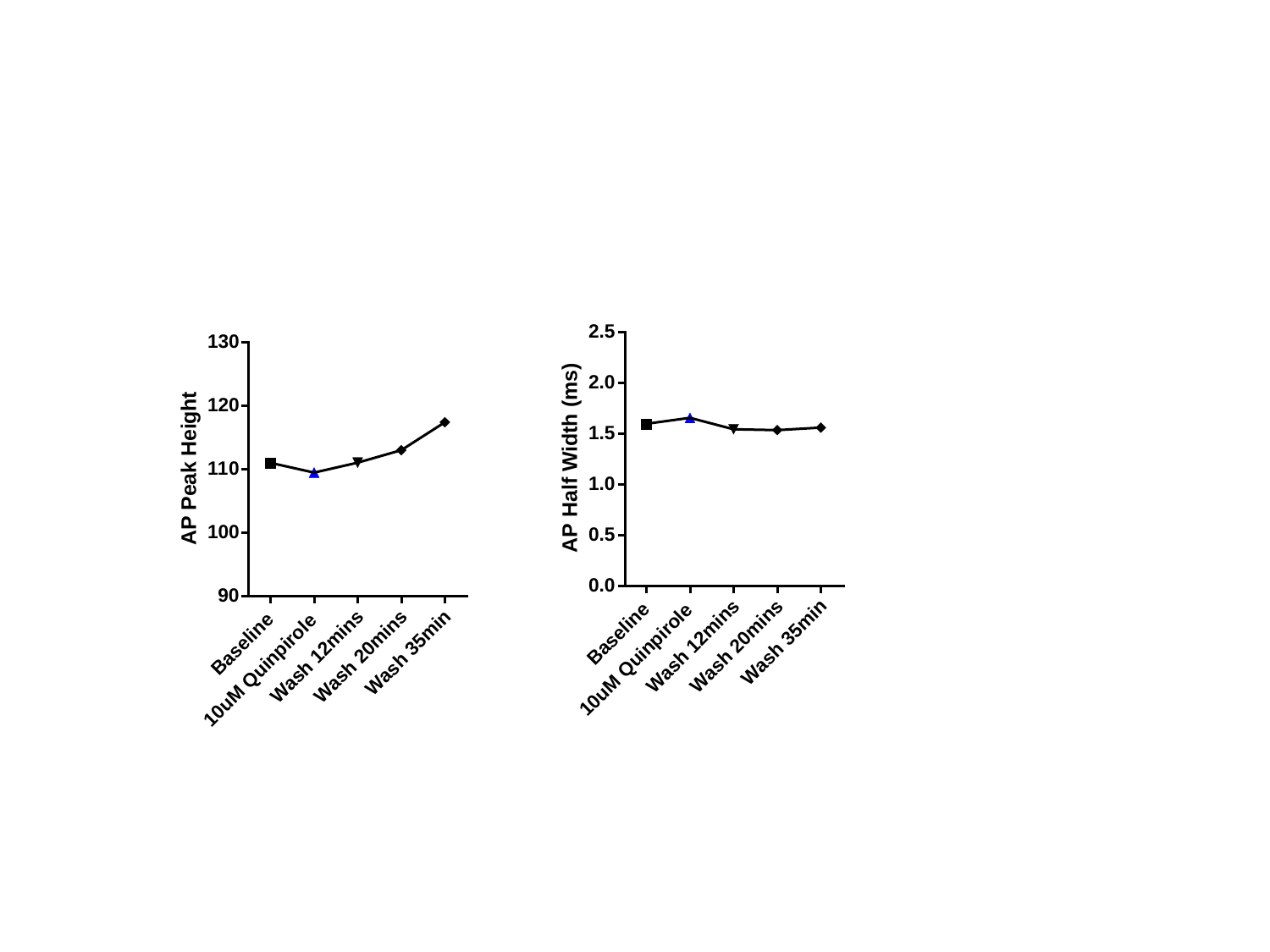

## Slide 42
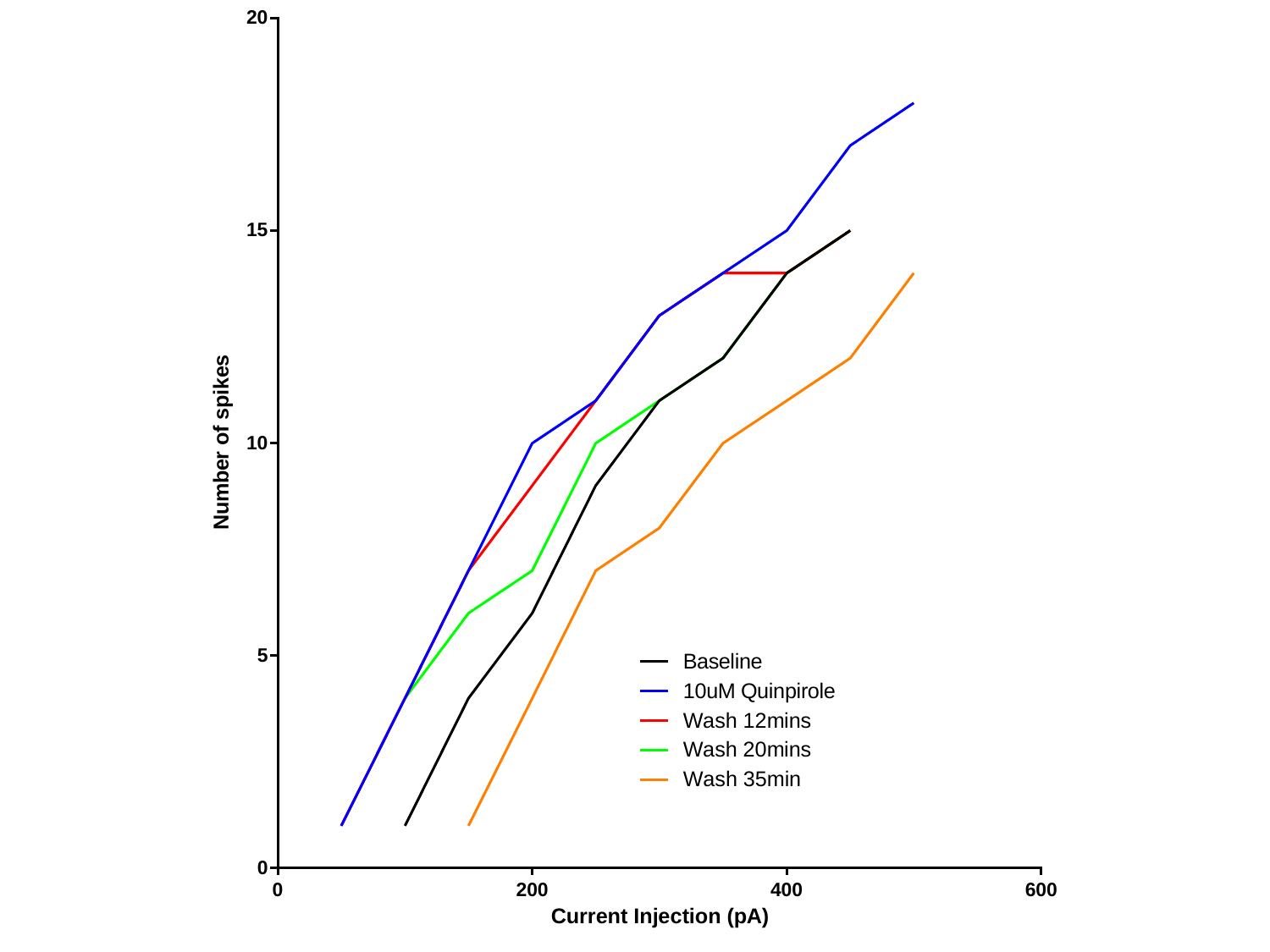

## Slide 43
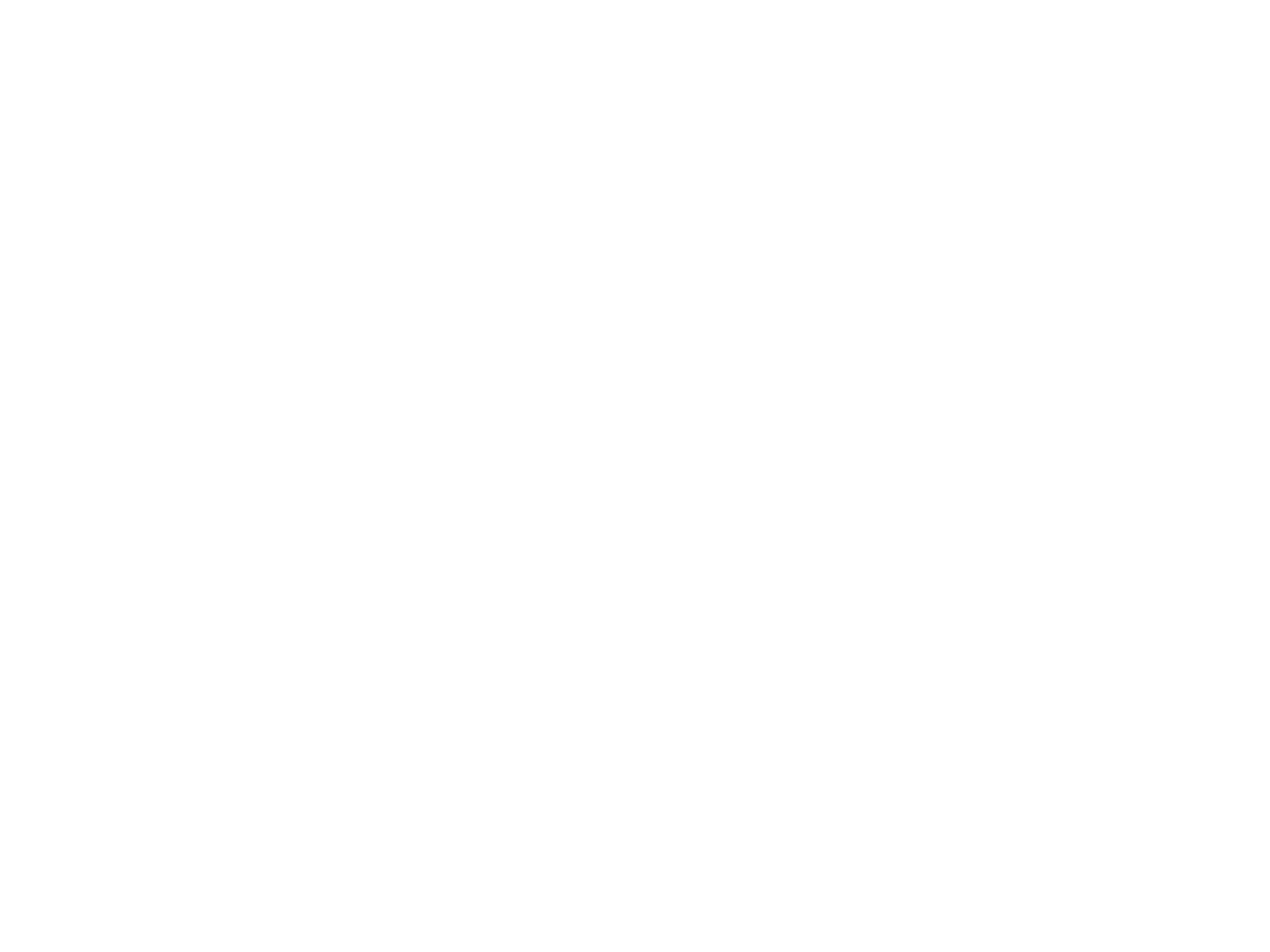

## Slide 44
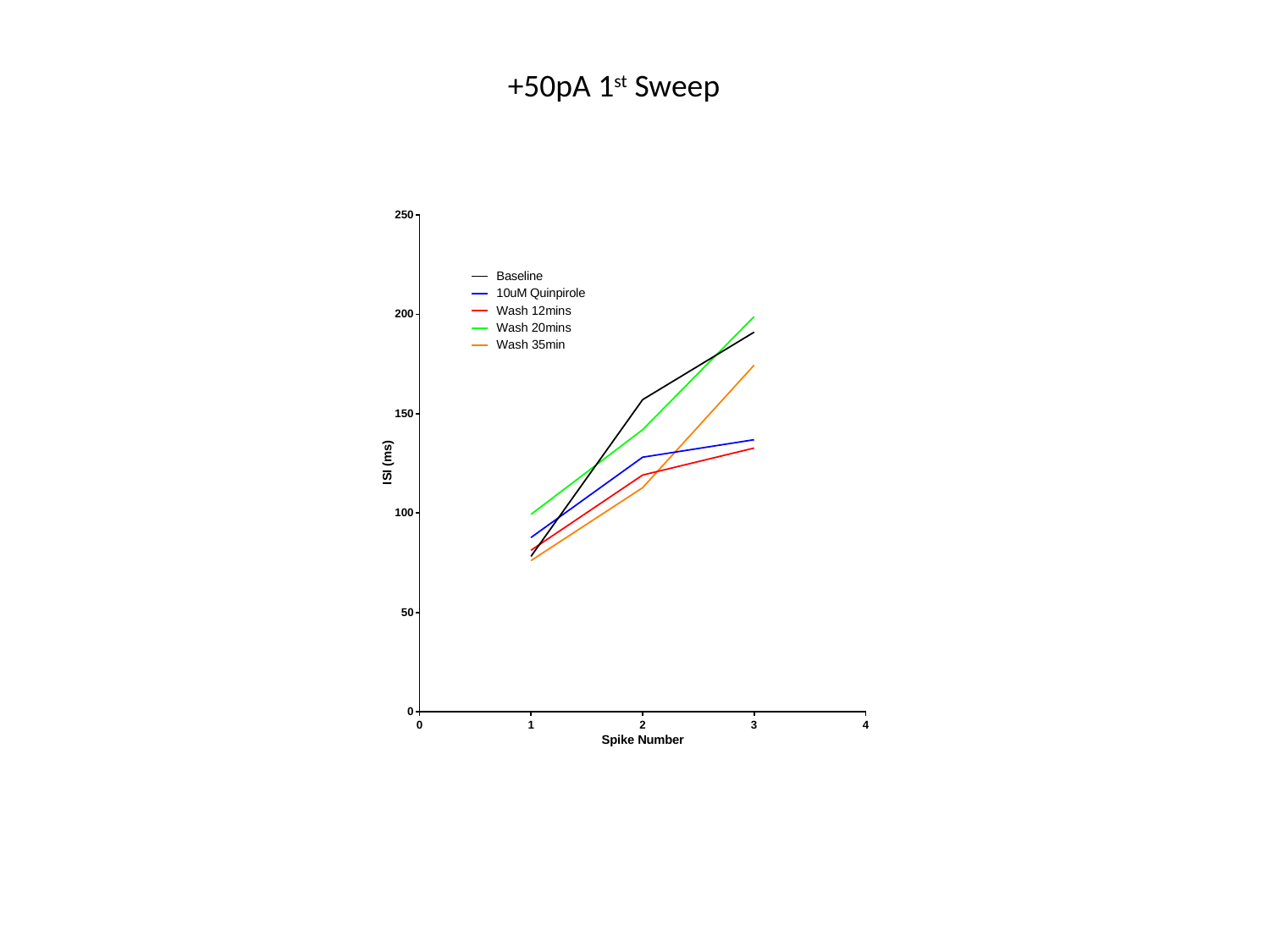

+50pA 1st Sweep

## Slide 45
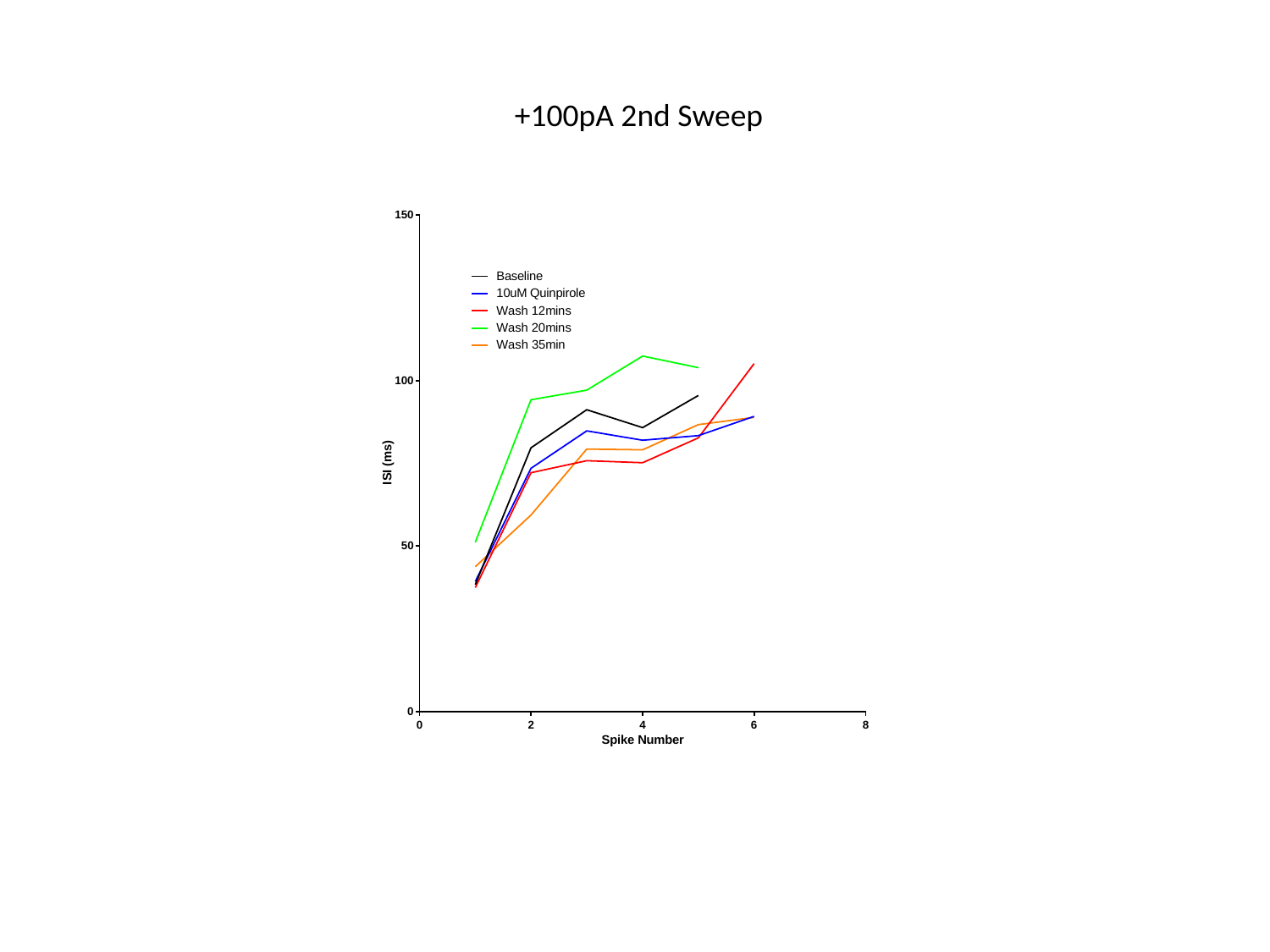

+100pA 2nd Sweep

## Slide 46
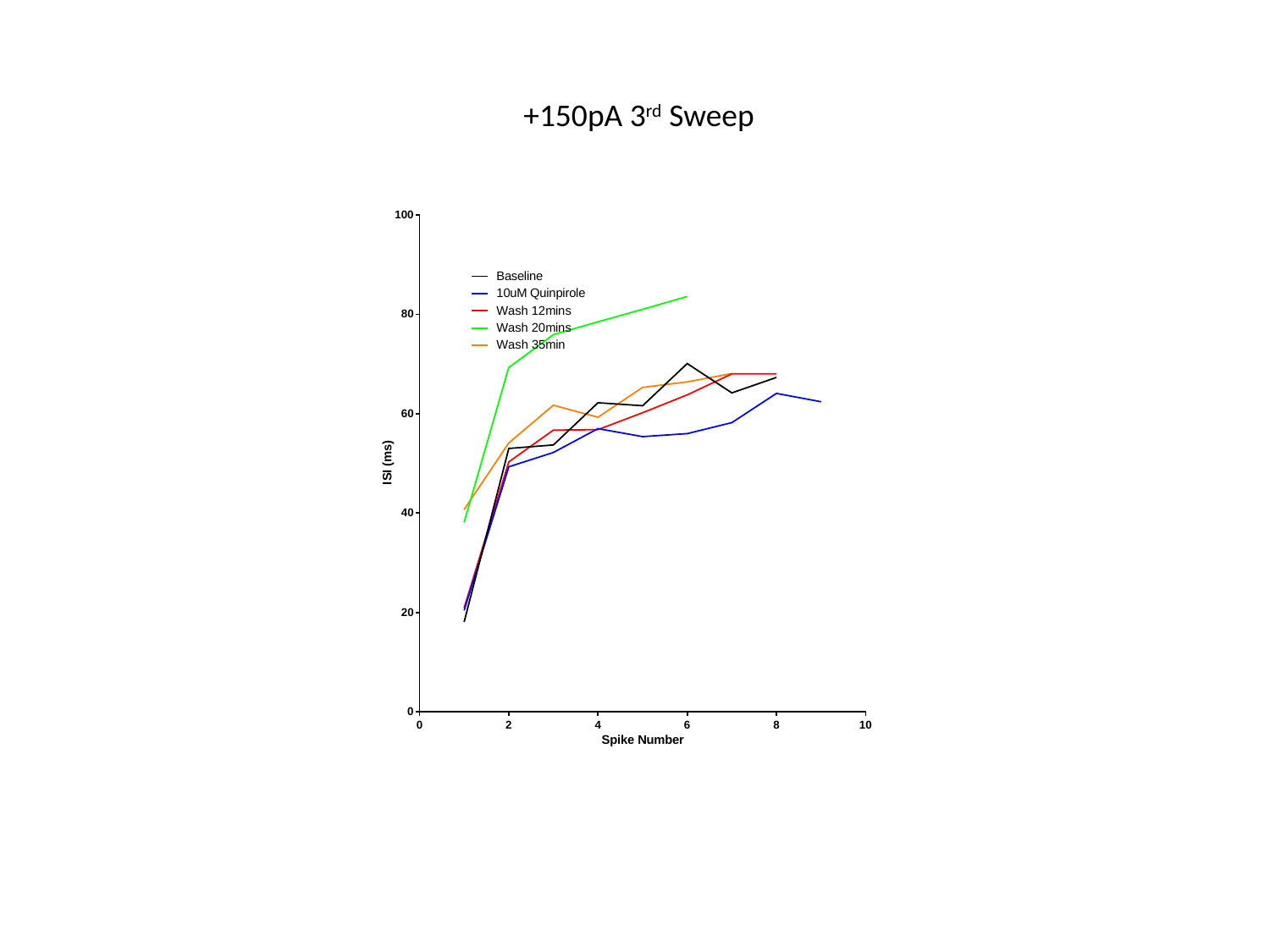

+150pA 3rd Sweep

## Slide 47
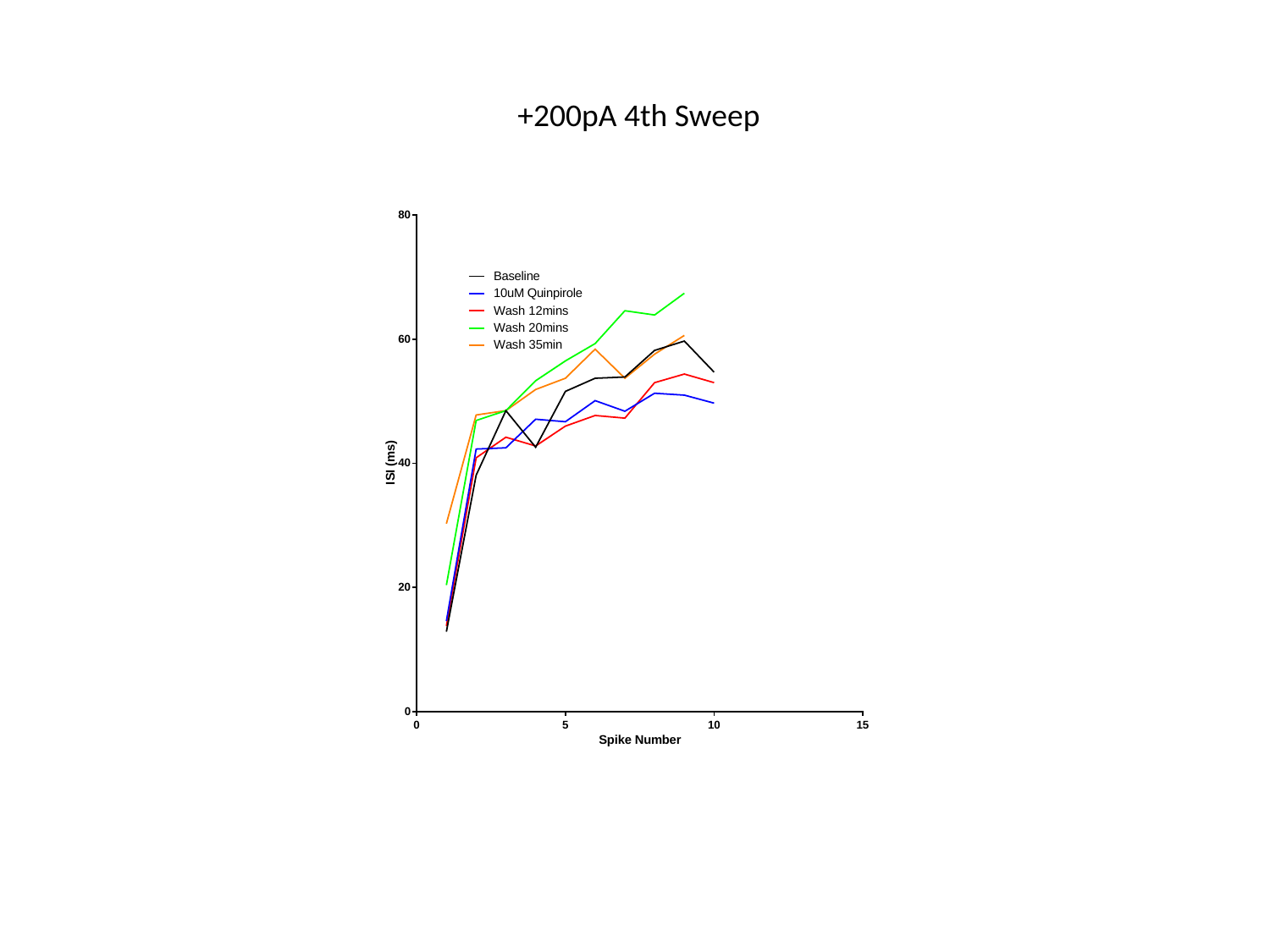

+200pA 4th Sweep

## Slide 48
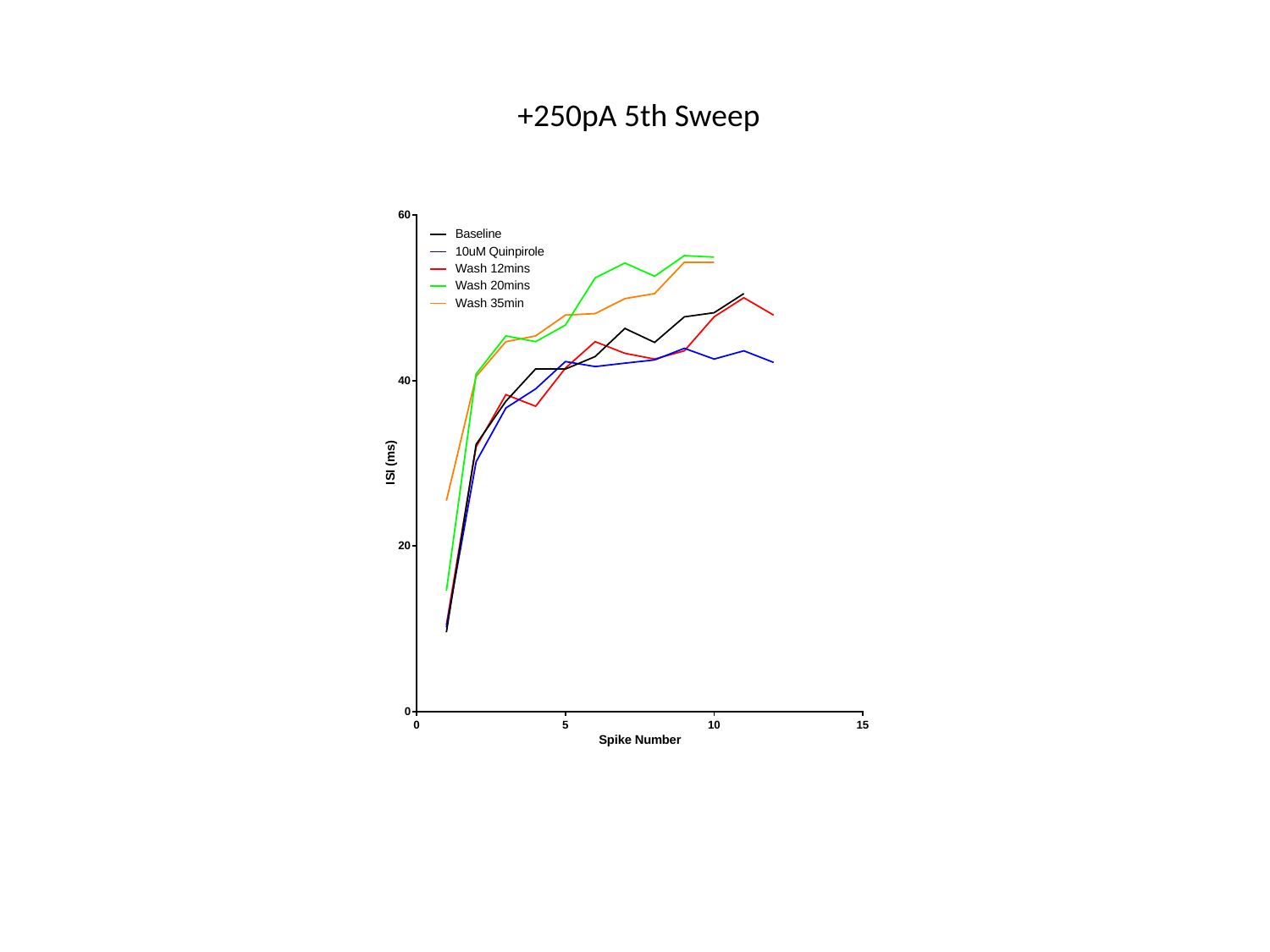

+250pA 5th Sweep

## Slide 49
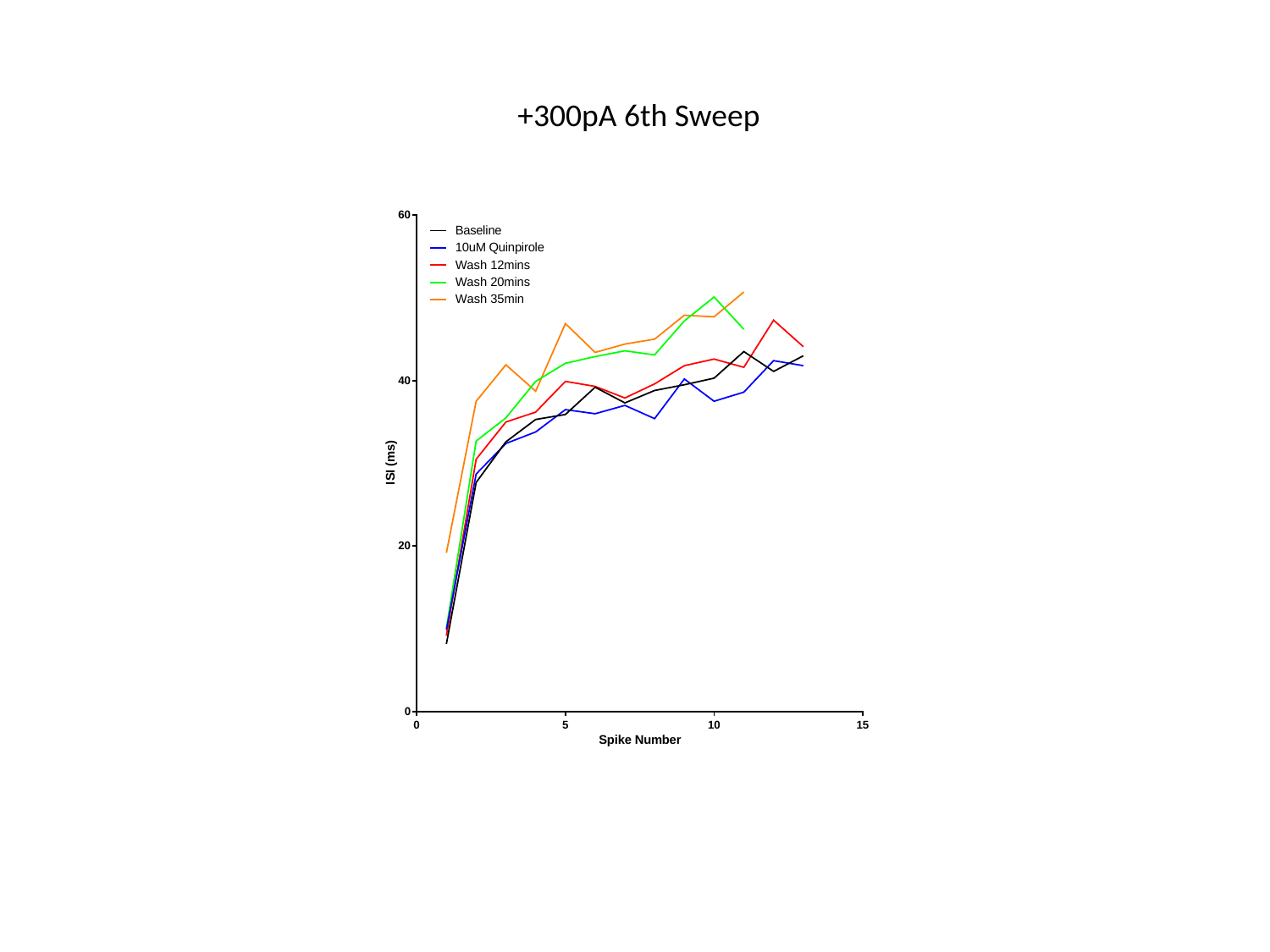

+300pA 6th Sweep

## Slide 50
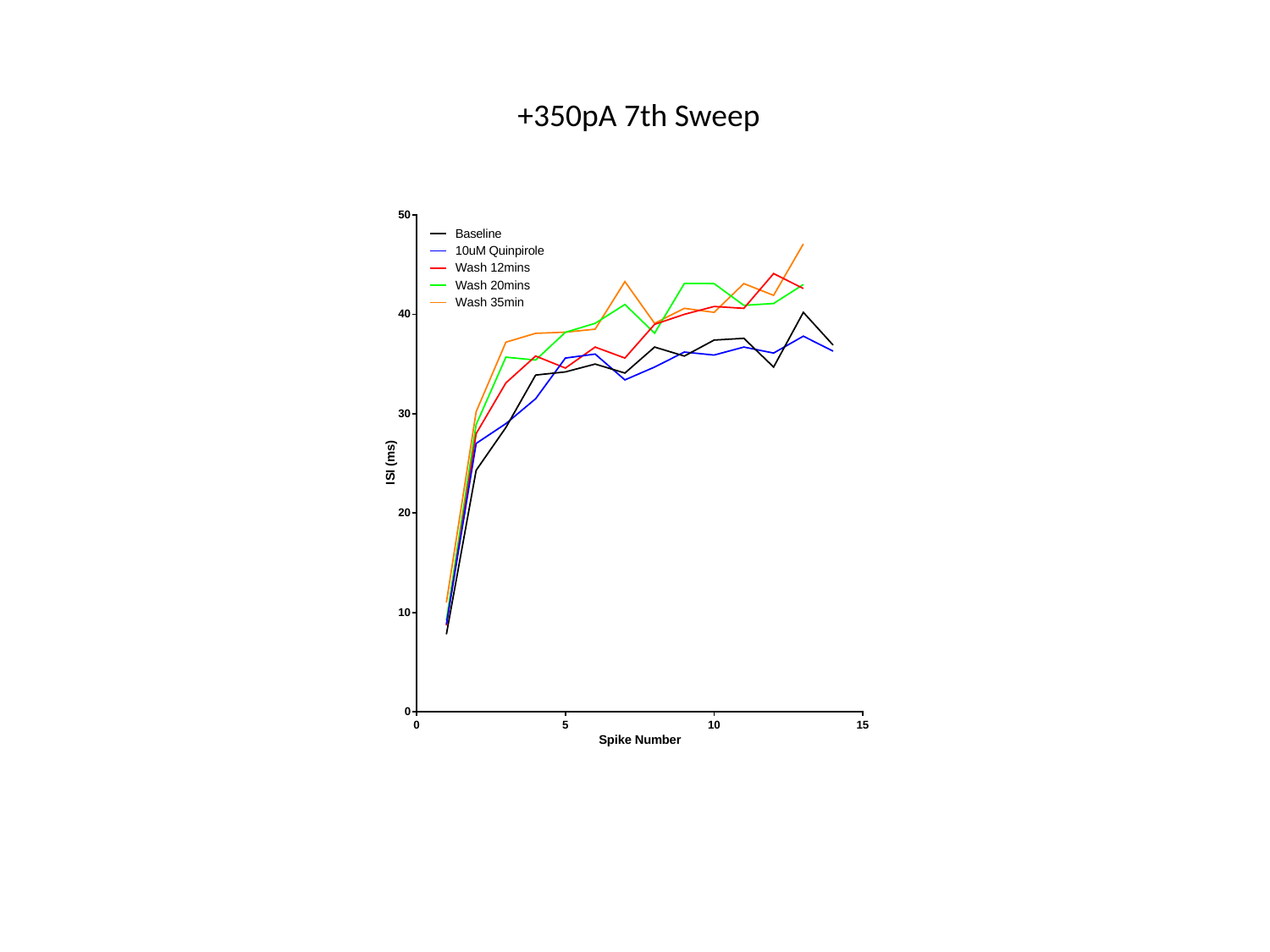

+350pA 7th Sweep

## Slide 51
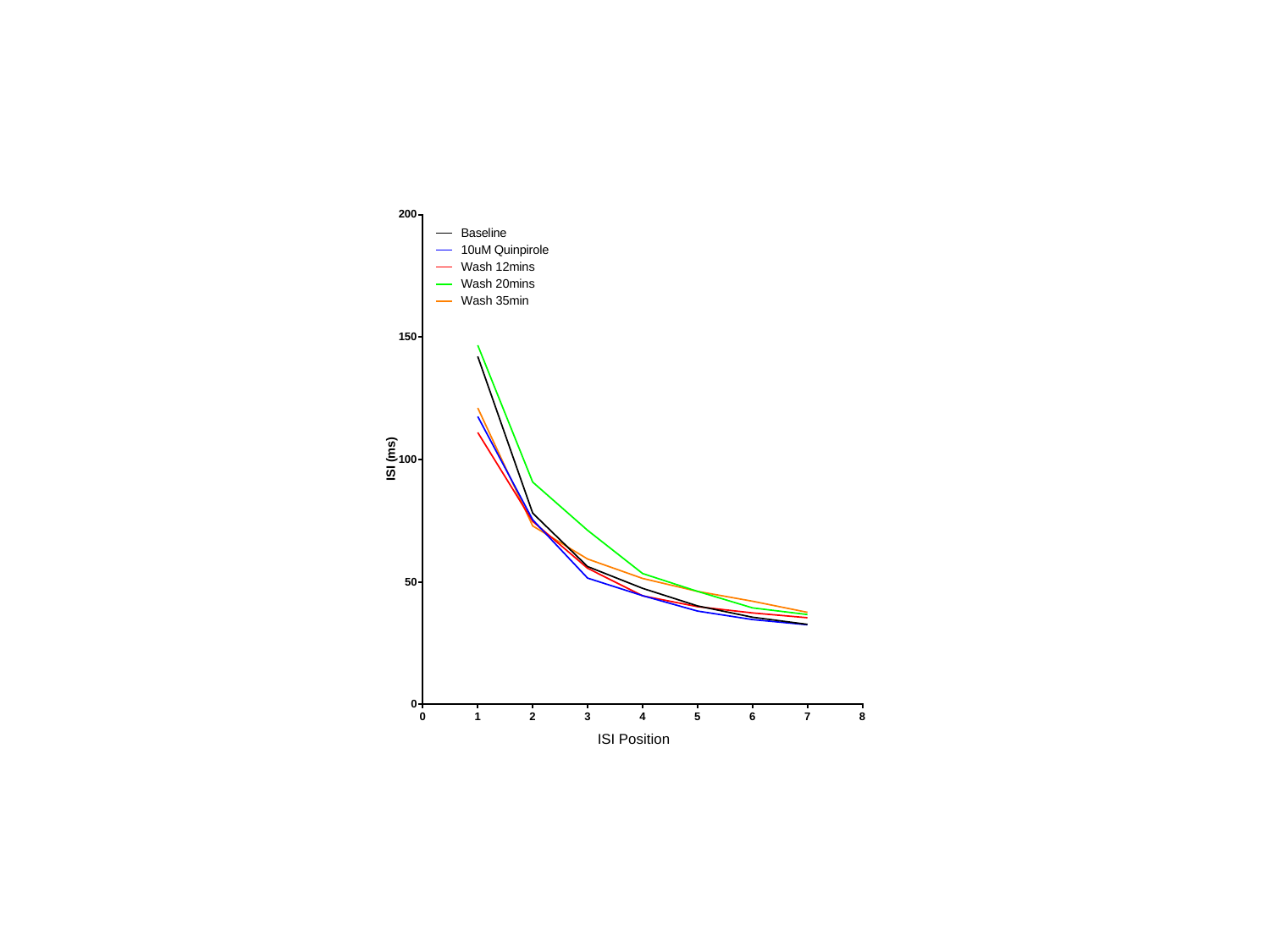

## Slide 52
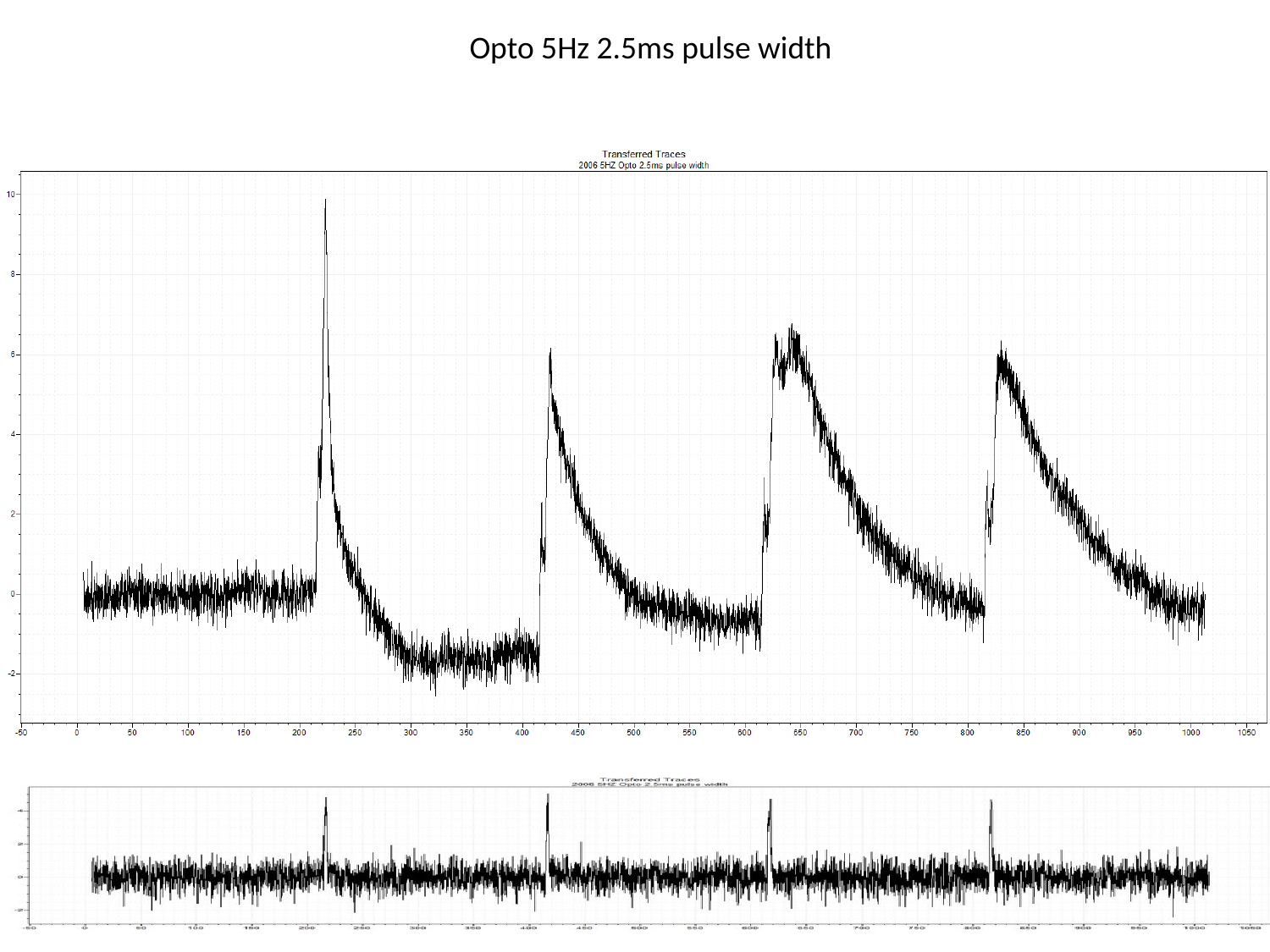

Opto 5Hz 2.5ms pulse width

## Slide 53
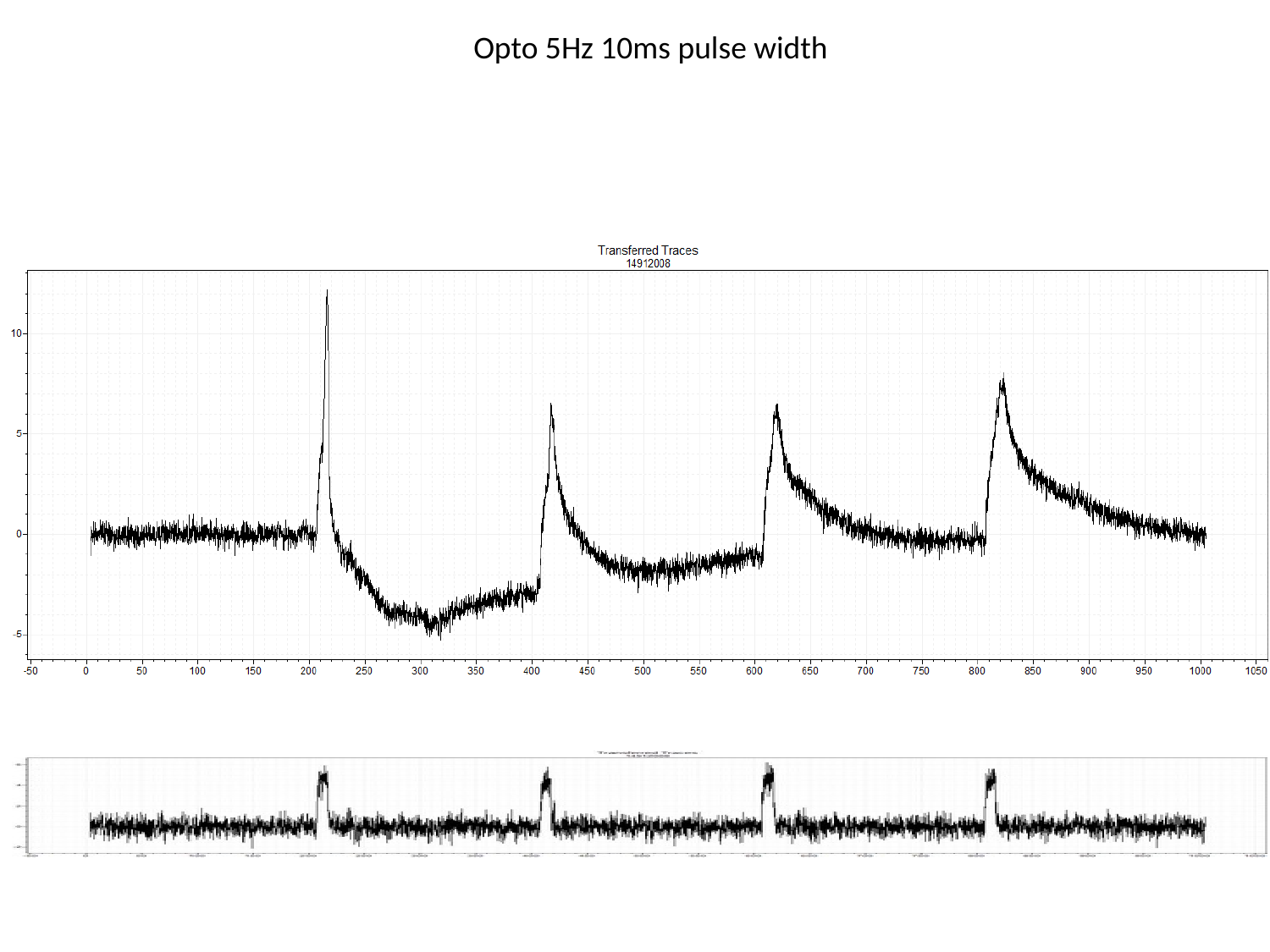

Opto 5Hz 10ms pulse width

## Slide 54
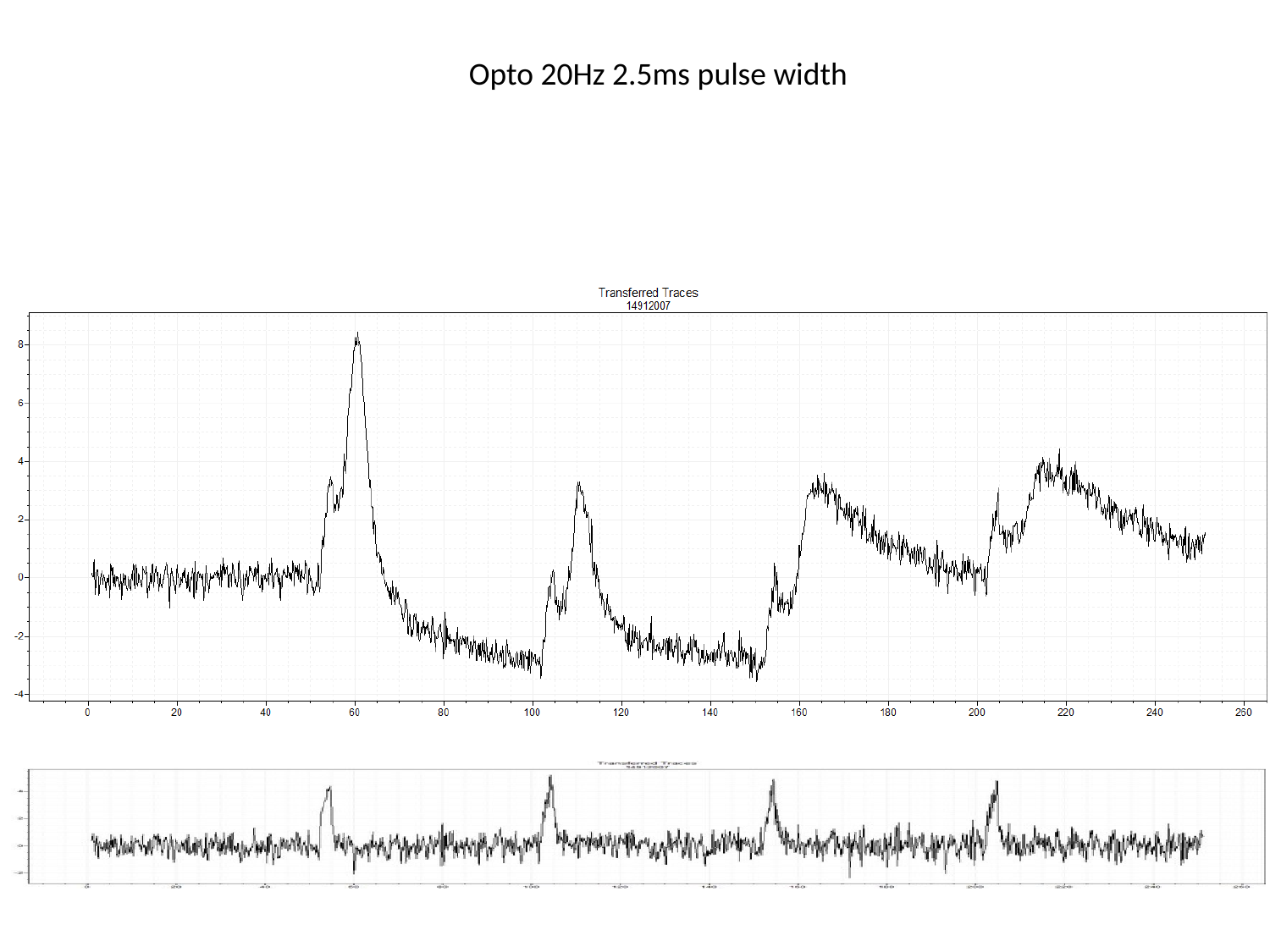

Opto 20Hz 2.5ms pulse width

## Slide 55
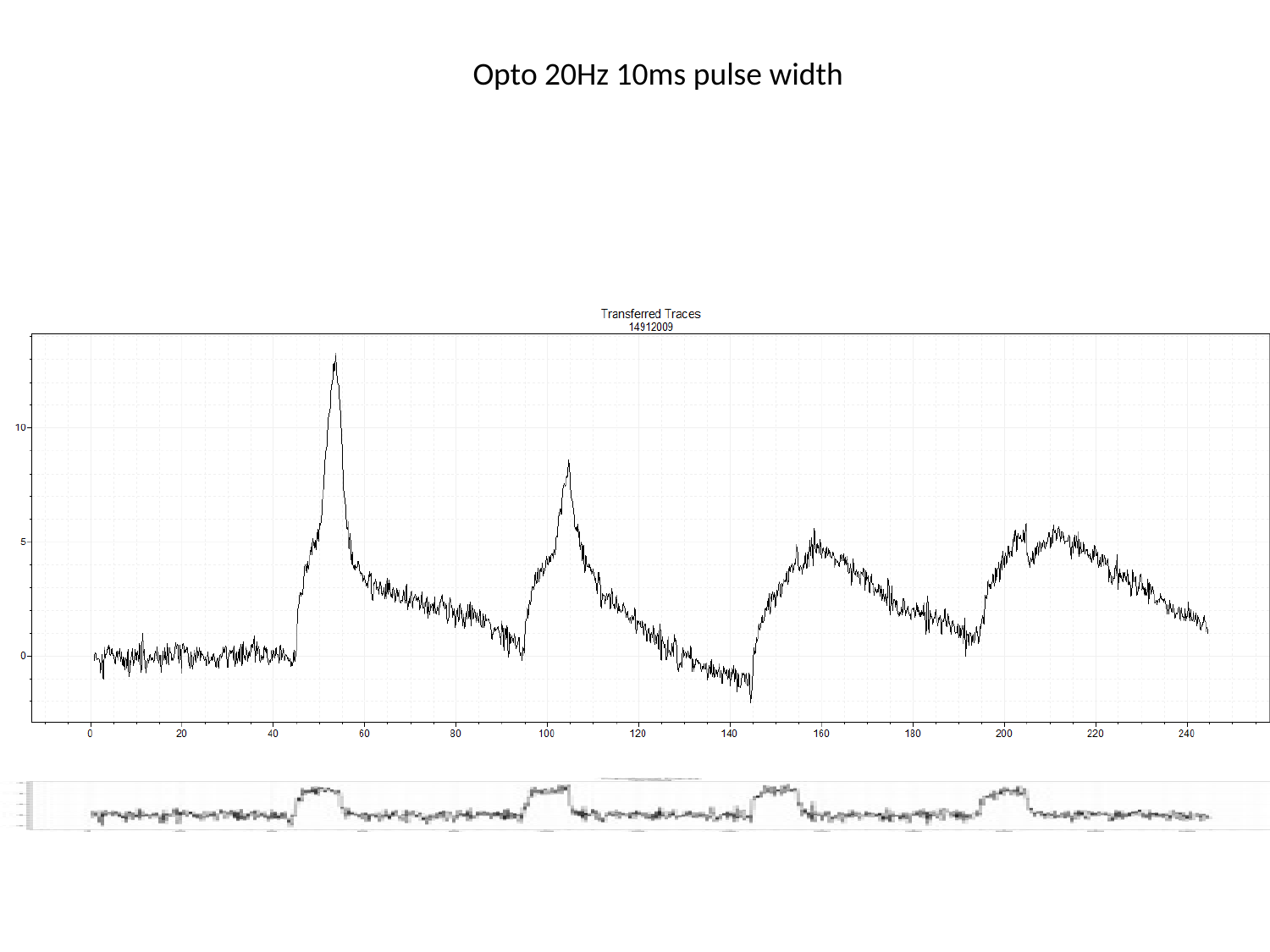

Opto 20Hz 10ms pulse width

## Slide 56
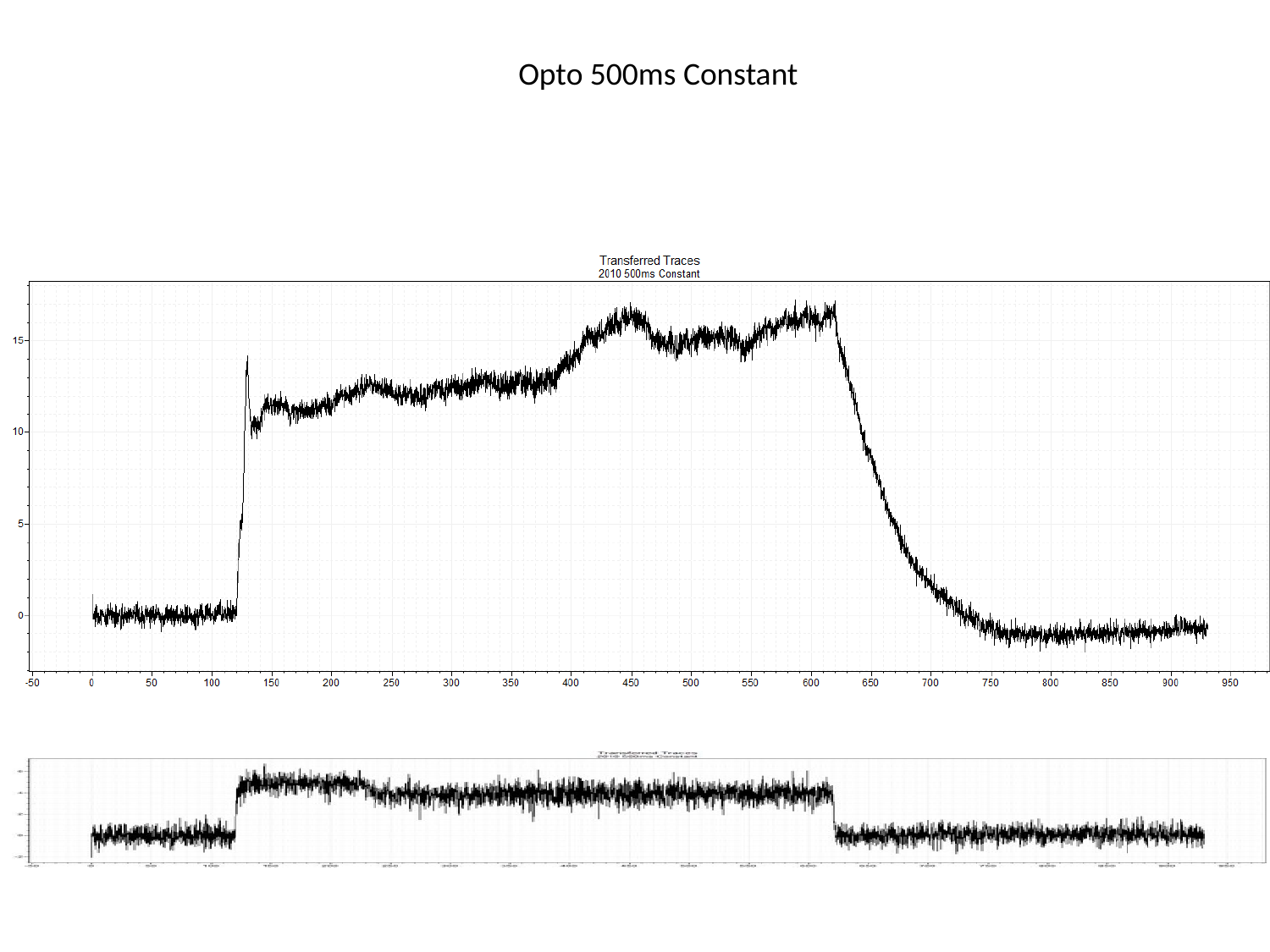

Opto 500ms Constant
